# Alteration of circulating miRNAs during myocardial infarction and association with lipid levels

**DOI:** 10.1093/labmed/lmad094

**Published:** 2023-12-04

**Authors:** Aybike Sena Ozuynuk-Ertugrul, Berkay Ekici, Aycan Fahri Erkan, Neslihan Coban

**Affiliations:** Department of Genetics, Aziz Sancar Institute of Experimental Medicine, Istanbul University, Istanbul, Turkey; Institute of Graduate Studies in Health Sciences, Istanbul University, Istanbul, Turkey; Department of Cardiology, Faculty of Medicine, Ufuk University, Ankara, Turkey; Department of Cardiology, Faculty of Medicine, Ufuk University, Ankara, Turkey; Department of Genetics, Aziz Sancar Institute of Experimental Medicine, Istanbul University, Istanbul, Turkey

**Keywords:** miR-126-3p, miR-210-3p, let-7g-5p, biomarker, coronary artery disease, myocardial infarction

## Abstract

**Background:**

Increasing mortality and morbidity of coronary artery disease (CAD) highlight the emerging need for novel noninvasive markers such as circulating microRNAs (miRNAs).

**Objective:**

To evaluate the circulating levels of miR-126-3p, miR-210-3p, let-7g-5p, and miR-326, and their associations with known contributors to CAD, in CAD subgroups.

**Methods:**

We divided the cohort into 4 groups: non-CAD controls (≤30% stenosis; n = 55), and patients with stable angina pectoris (SAP; n = 48), unstable AP (UAP; n = 46), and myocardial infarction (MI; n = 36). The circulating levels of miR-126-3p, miR-210-3p, let-7g-5p, and miR-326 were determined using TaqMan Advanced miRNA Assays in serum specimens.

**Results:**

Circulating miR-126-3p levels were lower in the MI and UAP groups, compared with the non-CAD group, whereas miR-210-3p circulating levels were lower in the MI group than others. The levels of circulating let-7g-5p were shown to be useful for distinguishing UAP from MI, and there were substantial differences in circulating let-7g-5p levels between the UAP and MI groups. Moreover, lipid levels and ratios were lower in individuals with high circulating miR-126-3p and miR-210-3p levels.

**Conclusions:**

The study results suggest that circulating miR-126-3p, miR-210-3p, and let-7g-5p are differentiated between different clinical presentations of CAD and associated with lipid levels, which are important risk factors and determinants of CAD.

## Introduction

Atherosclerosis is a process that involves the accumulation of lipids, cells (macrophages, T lymphocytes, and smooth muscle cells), and the extracellular matrix after endothelial dysfunction.^1^ Coronary artery disease (CAD), which results from the formation of atherosclerotic plaque and chronic inflammation, is one of the cardiovascular diseases (CVD), and CVD remains the leading cause of mortality globally.^2^ CAD has a latency period of many years and has different clinical presentations, such as stable angina pectoris (SAP), unstable angina pectoris (UAP), and myocardial infarction (MI). Angina pectoris is defined as substernal chest pain, pressure, or discomfort typically exacerbated by exertion or emotional stress. In SAP and UAP, which are subgroups of angina pectoris, coronary arteries narrow partially.^3^ MI (or acute myocardial infarction [AMI]), another common result of atherosclerosis, blocks blood flow to the heart due to a blood clot in the atheroma plaque rupture area. As a result of MI, heart muscles, heart walls, and heart valves are severely damaged by arrhythmia.^4^

miRNAs are short noncoding RNAs, approximately 22 nucleotides in length, that regulate gene expression posttranscriptionally.^5^ Differentially expressed miRNAs are important in CAD pathogenesis.^6^ Circulating miRNAs are stably present in biological fluids such as serum, plasma, and urine; therefore, their use as prognostic and diagnostic noninvasive biomarkers is thought to be possible.^7^ Circulating miRNAs exhibit greater stability than cellular miRNAs, which can be attributed to their association with AGO2 or their presence within extracellular vesicles, such as exosomes and microvesicles.^8^ Previous studies, such as one by Altesha et al,^9^ which investigated the functionality of miRNAs as biomarkers, presented promising results. In other previous studies, such as one by Wang L et al,^10^ circulating levels of miRNAs such as miR-1, miR-133, and miR-208 were found to differ between patients with CAD and control individuals. The findings of several studies^11-13^ have shown that circulating miR-126-3p, miR-210-3p, let-7g-5p, and miR-326 appear to be differentially expressed miRNAs in patients with CAD compared with controls.

This study aimed to examine and compare the circulating levels of miR-126-3p, miR-210-3p, miR-326, and let-7g-5p in the serum specimens of patients with SAP, UAP, and MI, and controls in the non-CAD group. Also, we investigated the correlations between miRNA levels and well-known risk factors of CAD, such as serum lipid levels. Lastly, we evaluated the putative target genes of miR-126-3p, miR-326, miR-210-3p, and let-7g-5p by their possible contribution mechanism to CAD pathogenesis.

## Methods

### Study Population and Measurement of Risk Factors

In total, 185 study participants were recruited for and enrolled in the Cardiology Department of Ufuk University, Faculty of Medicine between September 1, 2015, and September 1, 2017, as defined previously.^14^ These individuals were sorted into the following groups as follows: non-CAD controls (≤30% stenosis; n = 55), SAP (n = 48); UAP (n = 46), and MI (n = 36). We obtained written informed consent from all participants. The specimen collection and analysis processes were conducted in compliance with the ethical guidelines of the Declaration of Helsinki and approved by the Institutional Review Board of Istanbul University (approval date: August 17, 2015; No. 1542). Measurement of CAD risk factors (fasting glucose, total cholesterol [TC], low-density lipoprotein cholesterol [LDL-C], fasting triglycerides [TG], high-density lipoprotein cholesterol [HDL-C], and hemoglobin) and MI markers (creatine kinase–myocardial band [CK-MB] and cardiac troponin I [cTnI]) were taken, and the TC/HDL-C, LDL-C/HDL-C, and log_10_(TG/HDL-C) ratios, as well as the triglyceride-glucose (TyG) index, were calculated, as previously described.^14^

### miRNA Extraction and Determination of miRNA Expression Levels by Quantitative RT-PCR

We performed miRNA isolation from the 100 µL of serum specimens from the individuals recruited in the study, using the miRNeasy Serum/Plasma Kit (QIAGEN) according to manufacturer instructions. The quantity and quality of the isolated miRNA specimens were assessed using the NanoDrop 2000 Spectrophotometer (Thermo Fisher Scientific). miRNA specimens were diluted into 7.5 ng/µL. Also, 4 µL (50 pM) of 5ʹ phosphorylated synthetic miR-39 from *Caenorhabditis elegans* (cel-miR-39), which is used as an exogenous control miRNA, was added to each specimen before the cDNA synthesis procedure (Applied Biosystems Life Technologies). The cDNA synthesis was performed using the TaqMan Advanced miRNA cDNA Synthesis Kit (Applied Biosystems Life Technologies), following the manufacturer-provided instructions. We performed quantitative real-time polymerase chain reaction (qRT-PCR) using the TaqMan Advanced MicroRNA Assay Kit (Applied Biosystems Life Technologies). qRT-PCR testing was performed with 2.5 µL cDNA using the LightCycler480 Real-Time PCR System (F. Hoffman-La Roche).

We analyzed the resulting data using the comparative threshold cycle (Ct) method (2^−ΔΔCt^ method) and reported the results as relative quantification values. For normalization of miR-126-3p (assay ID: 477887_mir, assay name: hsa-miR-126-3p), miR-210-3p (assay ID: 477970_mir, assay name: hsa-miR-210-3p), miR-326 (assay ID:478027_mir, assay name: hsa-miR-326), and let-7g-5p (assay ID:478580_mir, assay name: hsa-let-7g-5p) expressions, we used cel-miR-39-3p (assay ID: 478293_mir, assay name: cel-miR-39-3p) as the exogenous control. Circulating levels of miR-126-3p, miR-210-3p, and cel-miR-39-3p were examined in serum specimens from the male and female subjects in the non-CAD (n = 55), SAP (n = 48), UAP (n = 46) and MI (n = 36) groups. Meanwhile, let-7g-5p and miR-326 levels were examined in the serum specimens of male subjects (non-CAD group, n = 20; SAP, n = 19; UAP, n = 14; and MI, n = 26).

### Bioinformatic Analysis

We utilized the miRWalk, miRDB, miRMap, RNA22, and DIANA MicroT-CDS databases to determine putative miRNA target genes.^15-19^ The miRNA-target gene interaction pairings were chosen if at least 2 of the 5 databases predicted them. The Database for Annotation, Visualization, and Integrated Discovery (DAVID; version 6.8) was used to perform a functional analysis of the target genes to deduce their putative functions.^20^ Further, we used Enrichr, a web-based tool, to perform gene ontology (GO) annotations on the miRNA target genes we had obtained.^21^ ClueGO software was utilized for the pathway analysis of the putative target genes of the miRNAs using the Kyoto Encyclopedia of Genes and Genomes (KEGG) database.^22^ The relevancy of a biological pathway/term to the predicted miRNA target genes was determined using the Fisher exact test. The corrected *P* values of significant pathways/terms were <.05 (Benjamini-Hochberg procedure). Target genes associated with CVD were obtained from the Genetic Association Database (GAD) (update from August 2014; accessed March 2020) and Malacards database^23^ (version 1.05; accessed March 2020), as previously described.^24^

### Statistical Analyses

Categorical variables were analyzed using χ^2^ testing. The ANOVA test was used for the comparison of continuous variables between groups. Due to skewed distributions of HDL, TG, HbA1c, and fasting glucose parameters, we used the nonparametric Kruskal-Wallis test for these variables. Categorical variables were reported as percentages, whereas quantitative variables were presented as mean (SD) values. We performed Kruskal-Wallis testing to analyze the difference in serum miRNA expression between groups. Spearman rank correlation analysis was used to examine the correlations between miRNAs and biochemical variables. For the formation of tertile groups (T1, T2, and T3), we divided the study population into 3 equal groups, using 33.3th and 66.6th percentile values of circulating miRNA levels as cut-off values for each miRNA separately. The analyses using tertile groups were conducted using T1 and T3. T1 of miRNAs represents individuals with low expression of miRNAs, whereas the T3 group was formed with individuals with high expression of miRNAs. We used the ROC curve to determine the ability to discriminate between groups and also calculated the AUC. Statistical analyses were conducted using SPSS (version 23.0; SPSS) and GraphPad Prism (version 8; GraphPad Software). For all tests, *P* <.05 was evaluated as being statistically significant.

## Results

### Baseline Characteristics of Study Subjects

Of the enrolled 185 subjects, 55 were in the non-CAD control group (mean age [SD], 65.4 [8.9] years), 48 had been diagnosed with SAP (mean age, 52.0 [5.7] years), 46 had been diagnosed with UAP (mean age, 62.2 [12.8] years), and 36 had been diagnosed with MI (mean age, 54.0 [8.5] years). The baseline characteristics of the groups are shown in Supplemental Table S1. CAD family history significantly differed among groups, in addition to the levels of serum lipids, fasting glucose, CK-MB, and cTn-I. The prevalence of type 2 diabetes mellitus (T2DM), obesity, and hypertension was not different among the groups. Also, the frequencies of lipid-lowering drug usage, current smoking status, and use of antidiabetic drugs were not significantly different between groups (Supplemental Table S1).

### Expression Levels of Circulating miR-126-3p, miR-210-3p, miR-326, and let-7g-5p

The circulating levels of miR-126-3p and miR-210-3p were successfully determined in 171 individuals (non-CAD, n = 53; SAP, n = 44; UAP, n = 42; and MI, n = 32); let-7g-5p and miR-326 levels were determined in 74 male subjects (non-CAD, n = 18; SAP, n = 18; UAP, n = 12; and MI, n = 26) using qRT-PCR. The expression levels of miR-126-3p and miR-210-3p in 14 individuals, as well as let-7g-5p and miR-326 levels in 5 men, could not be determined due to issues with the quality of their RNA specimens.

**Figure 1.**
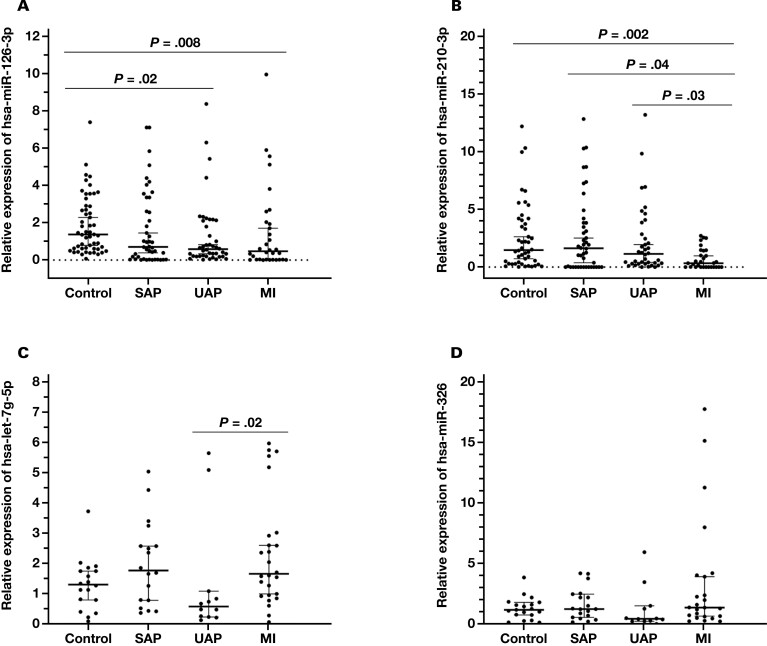
Circulating levels of miR-126-3p, miR-210-3p, miR-326, and let-7g-5p. A, The circulating levels of miR-126-3p were significantly different in the myocardial infarction (MI) and unstable angina pectoris (UAP) groups, compared with control individuals from the non–coronary artery disease (CAD) group. In pairwise comparisons other than UAP vs control and MI vs control, the circulating levels of miR-126-3p were not significantly different. B, In pairwise comparisons, the circulating levels of miR-210-3p were significantly different in the MI group compared with the UAP, SAP, and non-CAD control groups. C, In pairwise comparisons, the circulating levels of let-7g-5p were significantly different in the MI group compared to the UAP group. D, The circulating levels of miR-326 were not significantly different between groups.

Pairwise comparisons were made in the non-CAD, SAP, UAP, and MI groups. The levels of miR-126-3p expression were to be significantly decreased in the MI group, compared with the non-CAD group (*P* = .008; Figure 1A). Also, the expression of miR-126-3p was lower in the UAP group, compared with non-CAD controls (*P* = .02; Figure 1A). When we analyzed circulating miR-210-3p levels, we discovered that the expression levels were lower in the MI group, compared with those levels in the UAP, SAP, and non-CAD control groups (*P* = .03, *P* = .04, and *P* = .002, respectively; Figure 1B). When we compared the circulating levels of let-7g-5p between selected groups, we found that the levels were significantly differentiated between UAP and MI groups (*P* = .02; Figure 1C). There was no statistically significant difference between groups regarding circulating miR-326 levels (Figure 1D).

### Clinical Presentation and Low and High Expressions of miRNA

Next, we divided the study population into tertiles as T1, T2, and T3, according to the expression levels of miRNAs, to better identify the associations and to eliminate the bias that takes root from outliers. The clinical presentation of CAD was examined in high and low expression tertiles of miR-126-3p, miR-210-3p, and let-7g-5p, and the distributions of patients between groups were significantly different (Table 1). Moreover, the SAP, UAP, and MI groups were predominantly in the low expression tertile of miR-126-3p, compared with controls in pairwise comparisons (Table 1). These results were also statistically significant in the logistic regression analysis adjusted to age and sex (Table 1).

**Table 1.** High and low expression tertiles of miRNAs and clinical presentations of CAD

Variable	Group, % (No.)				χ2 *P* value	Multivariate logistic regression [OR (95% CI); *P* value]^a^			
	Non-CAD	SAP	UAP	MI		Non-CAD vs SAP	Non-CAD vs UAP		Non-CAD vs MI
Low miR-126-3p expression	23.3 (7)	53.5 (16)^b^	62.1 (18)^c^	64.0 (16)^d^	**.006**	Reference: 1.00			
High miR-126-3p expression	76.7 (23)	46.7 (14)	37.9 (11)	36.0 (9)		0.014 (0.001-0.351); ***P* = .01**	0.042 (0.007-0.263) ***P* = .001**		0.040 (0.004-0.377) ***P* = .005**
	Non-CAD	SAP	UAP	MI		Non-CAD vs MI	SAP vs MI	UAP vs MI	Non-MI vs MI
Low miR-210-3p expression	36.4 (12)	46.9 (15)	50.0 (11)	78.9 (15)^e-h^	**.03**	Reference: 1.00			
High miR-210-3p expression	63.6 (21)	53.1 (17)	50.0 (11)	21.1 (4)		0.046 (0.005-0.394) ***P* = .005**	0.220 (0.039-1.232) *P* = .09	0.575 (0.111-2.696) *P* = .51	0.230 (0.067-0.797) ***P* = .02**

	Non-CAD	SAP	UAP	MI		Non-MI vs MI^i^			
Low let-7g-5p expression	66.6 (6)	42.9 (6)	80.0 (8)	25.0 (4)^j,k^	**.03**	Reference: 1.00			
High let-7g-5p expression	33.3 (3)	57.1 (8)	20.0 (2)	75.0 (12)		4.562 (1.182-17.608) ***P* = .03**			
	Non-CAD	SAP	UAP	MI					
Low miR-326 expression	40.0 (4)	41.7 (5)	80.0 (8)	38.9 (7)	.16				
High miR-326 expression	60.0 (6)	58.3 (7)	20.0 (2)	61.1 (11)					

CAD, coronary artery disease; MI, myocardial infarction; SAP, stable angina pectoris; UAP, unstable angina pectoris.

^a^Logistic regression analysis adjusted for age and sex.

^b^Control vs SAP: P = .02.

^c^Control vs UAP; P = .003.

^d^Control vs MI; P = .002.

^e^Control vs MI; P = .003.

^f^SAP vs MI; P = .02.

^g^UAP vs MI; P = .06.

^h^Non-MI vs MI; P = .01.

^i^Logistic regression analysis adjusted for age.

^j^UAP vs MI; P = .01.

^k^Non-MI vs MI; P = .02.

When the T1 and T3 of miR-210-3p were compared between groups, the MI group was found predominantly in the low-expression tertile, compared with the control, SAP, and UAP groups, in pairwise comparisons. Moreover, when individuals were compared according to MI status, patients without MI were more prevalent in certain high-expression tertile groups compared to patients with MI (Table 1). However, the differences between control and MI groups and non-MI vs MI were found to be statistically significant in the logistic regression analysis adjusted to age and sex (Table 1). Still, we found the high expression tertile of let-7g-5p to be more prevalent in the MI group compared to the UAP and non-MI groups (control, SAP, and UAP; Table 1). Also, high expression of let-7g-5p increased the risk for MI in the logistic regression analysis adjusted for age (Table 1). There was no statistically significant difference between clinical presentation and tertiles of miR-326 (Table 1).

### The Diagnostic Accuracy of miR-126-3p, miR-210-3p, and let-7g-5p

**Figure 2.**
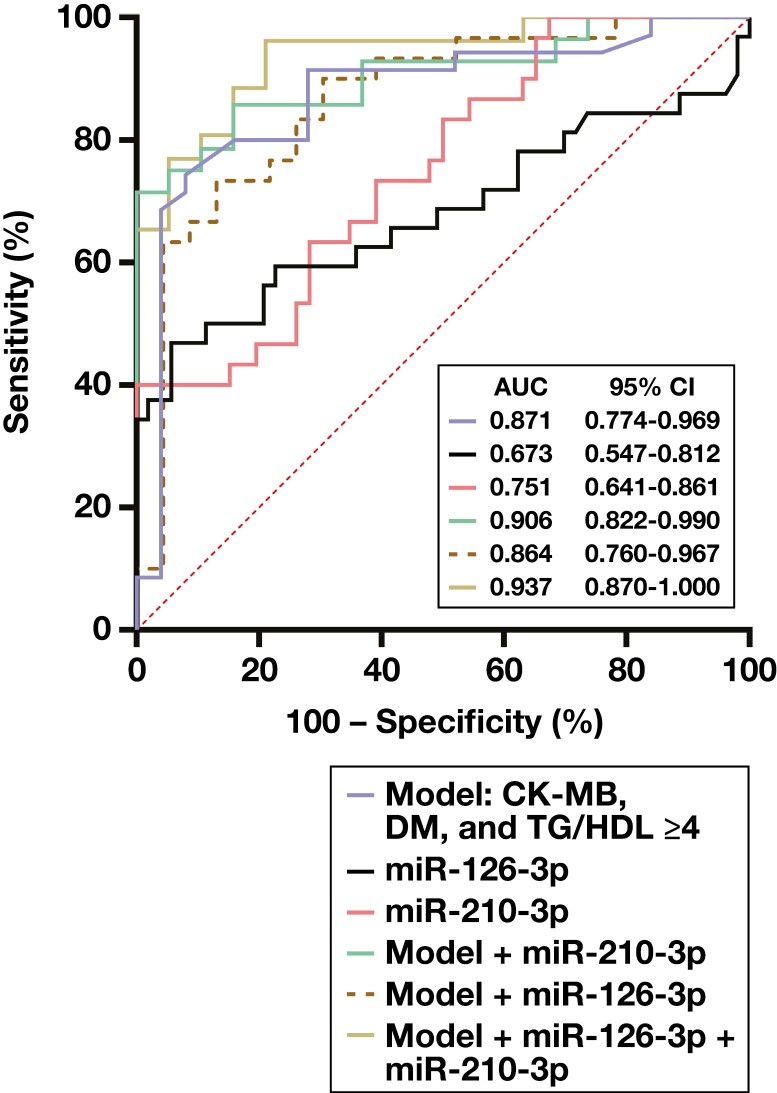
Sensitivity and specificity of circulating miR-126-3p and miR-210-3p. ROC curve analyses were performed to determine the discriminative ability of miR-126-3p and miR-210-3p among non–coronary artery disease (CAD) and myocardial infarction (MI) groups.

To evaluate the sensitivity and specificity of miR-126-3p, miR-210-3p, and let-7g-5p for diagnosing SAP, UAP, and MI, the AUC value was calculated using ROC curves (Supplemental Figure 1). As shown in Figure 2, miR-126-3p has a moderate power for distinguishing the MI group from the non-CAD group with the AUC value of 0.673 (95% CI, 0.547-0.812). The sensitivity and specificity of miR-126-3p for the MI group vs non-CAD controls were determined to be 59.4% sensitivity and 77.4% specificity, with a 0.60 cut-off. The AUC of miR-210-3p for the distinction of MI and non-CAD controls was found to be 0.751 (63.3% sensitivity and 71.7% specificity with 0.50 cut-off; *P* < .001; Figure 2).

We also performed ROC curve analyses using the crucial MI risk factors of TG/HDL ratio and presence of diabetes mellitus (DM), to better test the power of miRNAs. The cut-off value for TG/HDL ratio was determined to be ≥4.0, according to previous study reports, such as one by Chen et al.^25^ The AUC value was found to be 0.871 for the model of CK-MB, TG/HDL ratio ≥4, and DM (Figure 2). When the model and miR-210-3p were evaluated together, the discrimination of MI from non-CAD was better (AUC, 0.906; 95% CI, 0.822-0.990; Figure 2). The discriminating power was best in the model, including CK-MB, DM, TG/HDL ≥4, miR-210-3p, and miR-126-3p, together with the AUC value of 0.937 (Figure 2). When the diagnostic accuracy of let-7g-5p for distinguishing UAP from MI was evaluated using ROC curve analysis, the AUC value was found to be 0.776 (95% CI, 0.593-0.959), with 84.6% sensitivity and 75% specificity (cut-off, 0.85) (Supplemental Figure 1).

### Associations Between Clinical and Biochemical Parameters and Circulating miRNA Levels

**Figure 3.**
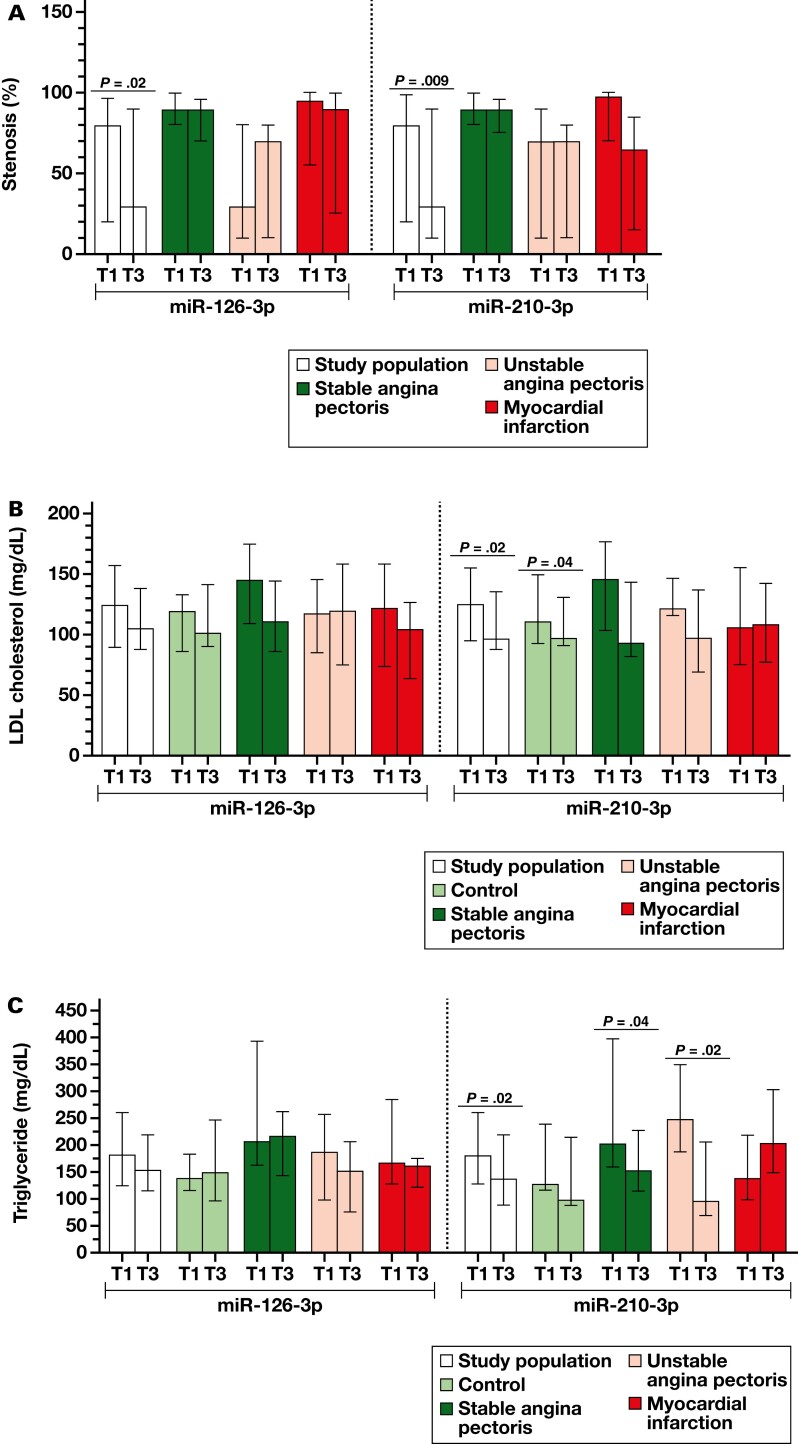

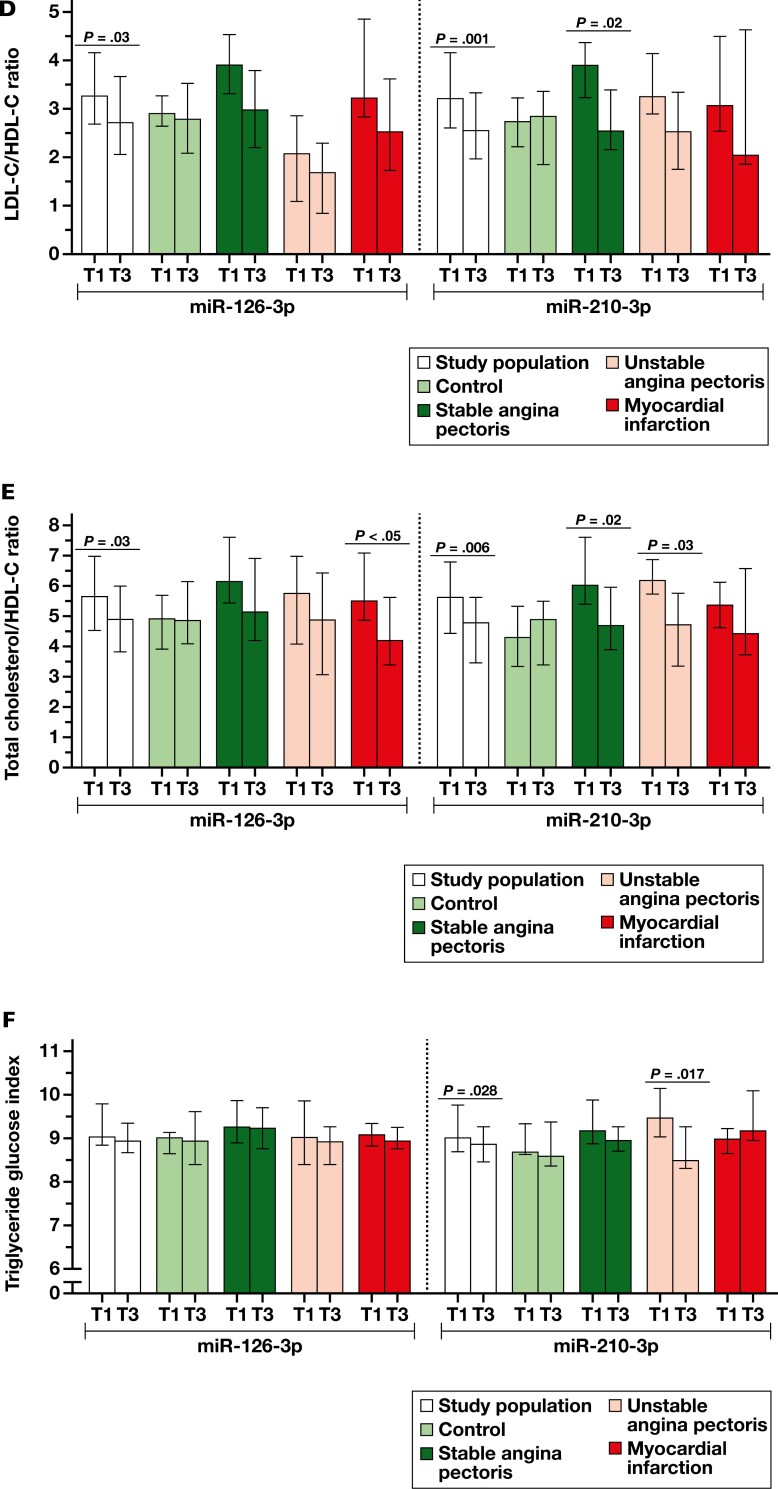
Expression tertiles of miR-126-3p and miR-210-3p are associated with clinical parameters. The stenosis percentage (A) is lower in the high tertiles of miR-126-3p and miR-210-3p. LDL-C (B), TG levels (C), LDL-C/HDL-C (D), TC/HDL-C ratios (E), and triglyceride-glucose (TyG; F) index were lower in the high miR-210-3p expression tertile in the subgroups. In the study population, LDL-C level and TC/HDL-C ratio were lower in the high miR-126-3p expression tertile.

The associations between clinical and biochemical parameters and tertile groups of miRNAs were evaluated in the study population, as divided into the control, SAP, UAP, and MI groups. We discovered that the stenosis percentage differs between T1 and T3 of miR-126-3p and miR-210-3p in the study population (Figure 3). Also, plasma lipid levels were associated with tertiles of miR-210-3p and miR-126-3p (Figure 3). In addition, a negative correlation was observed between CK-MB and miR-210-3p in the study population (*r* = −0.262; *P* = .007). Individuals in the low miR-210-3p tertile have shown significantly higher CK-MB levels (*P* = .01) and higher cTnI levels with borderline statistical significance (*P* = .08). The high miR-210-3p expression tertile had lower LDL-C levels in the study population and control groups (Figure 3), whereas the TG levels of the same tertile were lower in the study population, SAP, and UAP groups (Figure 3). Similarly, the low-expression tertiles of miR-126-3p and miR-210-3p have shown higher LDL-C/HDL-C and TC/HDL-C ratios in the subgroups (Figure 3). Also, miR-210-3p expression levels were significantly associated with the TyG index in the study population and UAP groups.

### In Silico Analyses of miR-126-3p, miR-210-3p, and let-7g-5p

**Figure 4.**
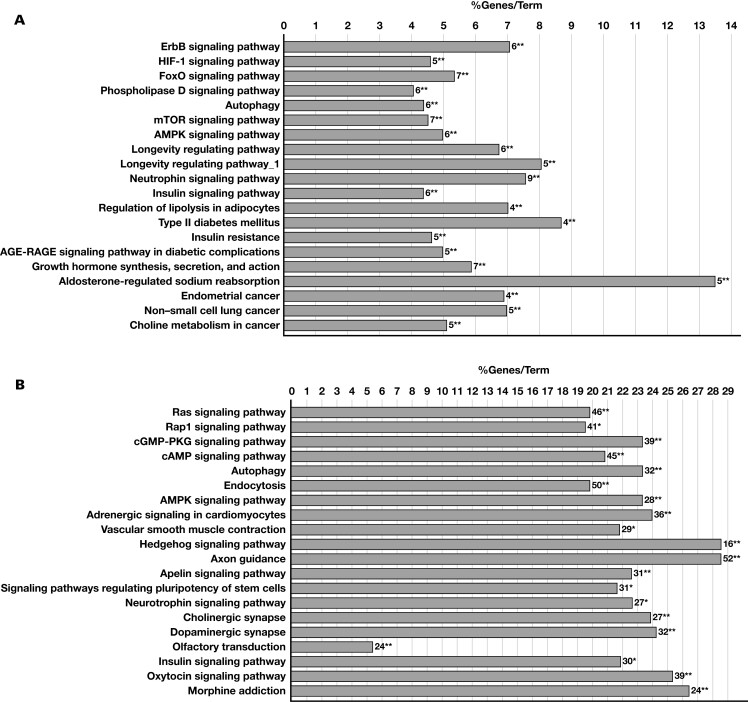
Kyoto Encyclopedia of Genes and Genomes (KEGG) pathway analyses. Pathway analyses were performed using the miRNA target genes we retrieved using the KEGG database terms by utilizing ClueGO. The Fisher exact test (adjusted using the Benjamini-Hochberg procedure) was used to assess the relevance of a biological pathway/term to the putative miRNA target genes. The top 20 pathways that putative target genes of miR-126-3p (A) and miR-210-3p (B) enriched were given. The numbers after the bars indicate the number of predicted targets of miRNAs in the pathway. *P ≤ .05; **P ≤ .01.

In bioinformatic analyses, 105, 244, 460, 1318, and 21 putative targets were found in DIANA MicroT-CDS, RNA22, miRMap, miRWalk, and miRDB databases, respectively, for miR-126-3p. In total, 158 of these genes were intersected in 2 of the databases, and 31 predicted genes were common in 3 databases. When the relationship of these putative targets with the CVD was evaluated, 49 and 3 of the target genes intersecting in the 2 databases were found to be associated in GAD and Malacards, respectively (Supplemental File). We discovered that 14 and 2 of the predicted target genes intersecting in 3 of the databases mentioned earlier herein were CVD-associated genes in GAD and Malacards, respectively (Supplemental File). Putative targets of miR-126-3p that intersected in 2 of the databases were found to be significantly enriched in the HIF-1 signaling pathway, FoxO signaling pathway, mTOR signaling pathway, AMPK signaling pathway, insulin signaling pathways, autophagy, and T2DM KEGG terms, along with others (Figure 4A; Supplemental File).

The bioinformatic analysis revealed 11044, 1203, 2164, 184, and 84 putative target genes in RNA22, miRMap, miRWalk, DIANA MicroT-CDS, and miRDB databases, respectively, for miR-210-3p. In total, 2221 of these target genes were intersected in 2 of the databases mentioned earlier herein, and 287 predicted genes were intersected in 3 databases. In the analysis of the DAVID bioinformatics tool, 612 predicted target genes in 2 databases were enriched in cardiovascular GAD disease class by 1.1-fold (adjusted *P* = .001) (Supplemental File). In all, 33 genes were associated with CHD, AMI, and MI in the Malacards database (Supplemental File). When the genes common in 3 of the 5 databases were evaluated for their relationship to CVD development, 87 and 4 were associated with CVD in GAD and Malacards, respectively (Supplemental File). In pathway analysis for the putative targets of miR-210-3p, we found that target genes were enriched significantly in CAD-related KEGG pathways such as vascular smooth muscle contraction, the insulin signaling pathway, the AMPK signaling pathway, and autophagy (Figure 4B; Supplemental File).

We found 3545, 13914, 2956, 1370, and 174 putative target genes for let-7g-5p in miRMap, RNA22, DIANA MicroT-CDS, and mirDB databases, respectively. In total, 4714 of these targets intersected in the results of at least 2 databases, and 1206 of these target genes are associated with CVD according to the GAD, whereas 62 are associated with CHD, AMI, and MI in the Malacards database. Moreover, in the analysis conducted using the DAVID bioinformatics tool, putative targets intersecting in 2 databases were enriched in GAD cardiovascular diseases and metabolic diseases by 1.1-fold change (adjusted *P* value [Benjamini-Hochberg method] = 4.2 × 10^−7^ and 4.2 × 10^−7^, respectively]. Also, putative target genes of the let-7g-5p were enriched in the MAPK signaling pathway, TGF-β signaling pathway, cGMP-PKG signaling pathway, and ABC transporters KEGG terms (*P* < .05 Benjamini-Hochberg corrected; Supplemental File).

## Discussion

**Figure 5.**
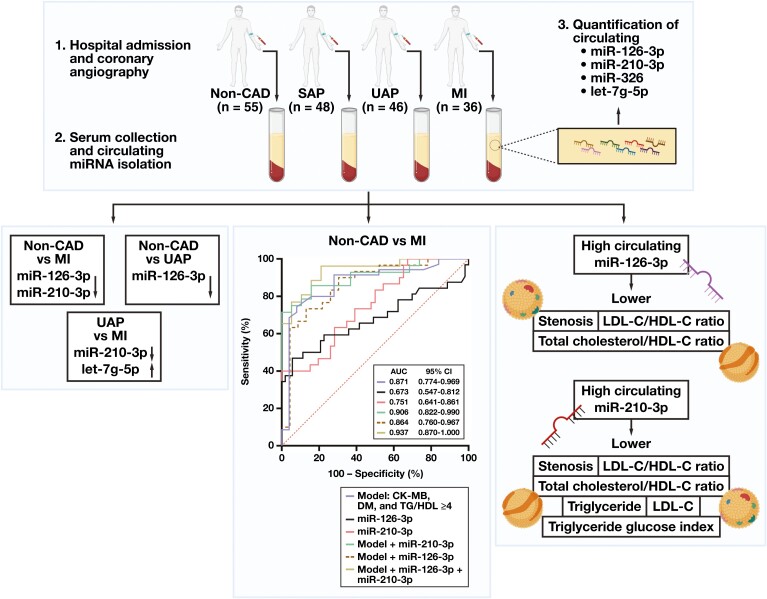
Graphical abstract. Individuals who underwent coronary angiography were grouped as controls not having non–coronary artery disease (CAD), as well as patients with stable angina pectoris (SAP), unstable angina pectoris (UAP), and myocardial infarction (MI). The circulating levels of miR-126-3p, miR-210-3p, let-7g-5p, and miR-326 were determined in serum specimens. The circulating levels of miR-126-3p were lower in the MI and UAP groups compared to the non-CAD group; circulating miR-210-3p levels were at lower levels in the MI group than others. The levels of let-7g-5p were significantly different between the UAP and MI groups. To evaluate the sensitivity and specificity of miR-126-3p, miR-210-3p, and let-7g-5p for diagnosing SAP, UAP, and MI, the AUC value was calculated using ROC curves. The AUC of miR-210-3p for the distinction of MI and non-CAD controls was found to be 0.751 (63.3% sensitivity and 71.7% specificity). The discrimination power was best in the model that includes creatine kinase–myocardial band (CK-MB), diabetes mellitus (DM), TG/HDL >4, miR-210-3p, and miR-126-3p with an AUC value of 0.927. Moreover, lipid levels, lipid ratios, and stenosis percentage were lower in individuals with high circulating miR-126-3p and miR-210-3p levels. The illustration was created via BioRender.com.

There is an ongoing investigation for the identification of noninvasive biomarkers that will make it possible to diagnose and elucidate the prognosis of CAD. In this study, we found that circulating miR-126-3p is downregulated significantly in patients with MI and UAP, compared with non-CAD controls. Also, circulating levels of miR-210-3p were downregulated significantly in the MI group, compared with the non-CAD control, SAP, and UAP groups. Circulating let-7g-5p was found at higher levels in the MI group, compared with the UAP group. Moreover, miR-210-3p and miR-126-3p were associated closely with serum lipid levels (Figure 5).

In the continuing search for a novel biomarker for CAD, miR-126-3p was one of the miRNAs examined. As previously demonstrated, the impact of miR-126 is influenced by the source of miR-126 and the specific mature strand (-3p or -5p) that is involved, as in atherosclerosis and angiogenesis processes.^26,27^ For instance, miR-126-3p mainly exhibits atheroprotective features on endothelial cells (ECs) through suppressing inflammation, whereas miR-126-5p primarily increases the proliferation of ECs and reducing atherosclerosis.^26^ These features highlight the functional complexity of this miRNA. As was comprehensively reviewed by Yu et al,^28^ miR-126-3p is associated with vascular or related diseases. Endothelial cells exhibit high levels of miR-126, which helps to preserve vascular integrity and promote angiogenesis.^29^

In a mouse model, genetic deletion of miR-126 resulted in the embryonic death of some mice related to vascular leakage, and the remaining mice had pathologies associated with angiogenesis deficiencies.^30^ Moreover, overexpression of miR-126 attenuated atherosclerotic plaque progression in APOE^-/-^ mice fed a high-fat diet.^31^ Circulating levels of miR-126-3p were examined in ACS groups, and related studies were concluded with rather contradicting results.^31-34^ In some previous studies, such as that by Wang et al,^33^ circulating levels of miR-126-3p decreased in patients with CAD, compared with controls, whereas others^31,32,34^ reported increased circulating levels.

In the present study, we determined that circulating miR-126-3p levels were lower in the MI and UAP groups, compared with the control group. Moreover, the high-expression tertile was more prevalent in the control group than in the SAP, UAP, and MI groups, and stenosis was lower in the high-expression tertile. In line with this finding, Fichtlscherer et al^35^ showed that circulating miR-126-3p levels are downregulated in patients with CAD compared to healthy controls.

miR-210-3p and miR-210-5p are the hypoxia-inducible miRNAs (hypoxamiRs) regulated by HIF-1α in the hypoxic environment.^36^ miR-210 regulates cell survival, proliferation, apoptosis, differentiation, and other physiological functions by controlling its downstream target genes after hypoxia. According to the results from recent studies, such as one by Diao et al,^37^ miR-210-3p is elevated in hypoxic cardiomyocytes and demonstrates cytoprotective features. In addition, Qiao et al^38^ showed that in macrophages, high miR-210-3p levels inhibit lipid accumulation and NF-κB-mediated inflammation by suppressing IGF2 in atherosclerosis. Also, improved plaque stability was established with the increased expression of miR-210 through APC inhibition and increased VSMC survival.^39^

miR-210-3p is evaluated as a potential biomarker in studies related to hypoxia, preeclampsia, cancer, and stroke, with better reliability and repeatability in circulation.^40-42^ From the perspective of CVDs, miR-210-3p levels in whole-blood specimens from 16 patients with CAD were increased, compared with 16 controls.^43^ In another study,^44^ plasma levels of miR-210-3p were upregulated in 9 patients with diabetes and CVD, compared with 6 patients who had diabetes but no CVD. In contrast, Qin et al^45^ showed that miR-210 levels were negatively correlated with the cardiac damage marker cTnl, a well-known marker upregulated in MI and cardiac damage. In line with Qin et al, in the present study we observed a negative correlation between miR-210-3p and CK-MB, which is also a well-known marker in MI, and higher CK-MB and cTnI levels in the low miR-210-3p tertile, compared with the high miR-210-3p tertile. In a microarray analysis conducted on patients with MI and healthy controls, Rincón et al^46^ demonstrated that circulating miR-210-3p levels are downregulated in patients with MI. In the present study, decreased circulating levels of miR-210-3p were observed in the MI group, compared with the SAP, UAP, and non-CAD groups; also, the stenosis percentage was lower in the high miR-210-3p tertile.

Zeller et al^47^ compared the circulating miR-210-3p levels on admission, and after 3 and 6 hours of admission, in 29 patients with ST-segment elevation MI (STEMI) and 63 controls with noncardiac chest pain (NCCP). These coauthors found that miR-210-3p levels are increased within 6 hours, although it was not specified whether there is a difference in miR-210-3p levels between the STEMI group and NCCP control group on admission. The gradual increase in the abundance of miR-210-3p highlights the importance of the specimen collection timeline. Other studies investigate the relation of miR-210 to the presence of CAD. However, we could not compare those findings with ours because the examined strand of the miR-210 (-3p or -5p) is not specified, which complicates the assessment of the results. To our knowledge, our study is the first in the literature to show the downregulation of circulating miR-210-3p in patients with MI compared to non-CAD controls, patients with SAP, and patients with UAP.

The molecular function and the target genes of let-7g-5p were previously studied in vivo and in vitro. Previous study findings demonstrated evidence for let-7g-5p reducing macrophage foam cell formation by inhibiting canonical and noncanonical NF-κB signaling pathways and reducing inflammatory and apoptotic responses. Also, in a few studies, the association between the circulating levels of let-7g-5p and the presence and severity of CAD was previously investigated. The circulating levels of let-7g-5p were associated with the segment stenosis score (SIS): this scoring system evaluates the severity of coronary atherosclerosis.^48^ In this study, it has been found that patients with severe atherosclerosis have lower circulating let-7g-5p.^48^ In line with this finding, serum let-7g-5p levels decreased during acute non−ST-elevated MI (NSTEMI), compared with a control group, in a study conducted by Mompeón et al.^49^

As a part of the HUNT (Nord-Trøndelag Healthy) study, Bye et al^50^ have shown that circulating let-7g-5p levels are decreased in patients with AMI, compared with healthy controls. In contrast, Velle-Forbord et al^13^ have shown that circulating levels of let-7g-5p are increased in patients with fatal MI compared to controls, as a part of the HUNT study. In the present study, although there was no difference between non-CAD controls and the MI group, the UAP group showed significantly lower circulating levels than the MI group. The contradicting results on the circulating levels of the examined miRNAs as a diagnostic marker indicate the need for analysis in larger study groups. The vast diversity and minimal overlap of aberrant miRNA expression patterns in different studies may be due to patient group, type of specimen, study design, various detection methods, and specimen collection procedures, as well as race and ethnicity.

The risk factors of CAD, such as low HDL-C, high LDL-C, and TG levels, were well defined among the lipid ratios, which were determined as CVD risk indicators. In addition to blood lipid levels, the LDL-C/HDL-C, TC/HDL-C, and TG/HDL-C ratios are thought to be markers of cardiovascular disorders because an imbalance in the cholesterol transported by protective and atherogenic lipoproteins might indicate a higher risk of CVD.^51^ Nam et al^52^ suggest that the TC/HDL-C and LDL-C/HDL-C ratios are more potent markers of CAD risk than LDL and HDL levels only. In a previous study,^53^ patients with CAD and high LDL-C levels had considerably lower levels of miR-126-3p. In line with this finding, although it did not reach statistical significance, the high miR-126-3p tertile showed lower LDL-C levels. In addition, the LDL-C/HDL-C and TC/HDL-C ratios were significantly lower in the high miR-126-3p tertile. Further, in this study, we found that LDL-C, TG, TC/HDL-C, LDL-C/HDL-C ratios, and TyG index are at lower levels in the high miR-210-3p tertile in several groups. Qiao et al^38^ demonstrated that miR-210-3p was negatively correlated with TG and TC levels, which is in line with our results. These findings point out the associations between the miRNAs and CAD risk factors and therefore highlight the importance of miRNAs as a potential target for the disease.

The results of our bioinformatics analyses have shown that putative target genes of miR-210-3p intersecting in 2 of the analyzed databases are significantly enriched in cardiovascular GAD disease class by 1.1-fold, whereas putative targets of let-7g-5p are enriched in metabolic diseases and CVD GAD classes significantly. The biological processes and the KEGG pathways that these putative targets have enriched are linked to the CAD pathological process. We found that putative targets of miR-210-3p and miR-126-3p are enriched in CAD-related KEGG pathways such as AMPK signaling, autophagy, and insulin signaling.

Several limitations must be taken into account. In this study, the size of the subgroups of CAD prevented us from considering the status of patients regarding use of lipid-lowering and antidiabetic drugs. Because miRNAs could also be bound to lipoprotein particles such as HDL, in future studies, status of usage of lipid-lowering drugs should be considered.

## Conclusions

Our study results indicate that miR-126-3p and miR-210-3p can discriminate individuals belonging in the non-CAD group from those with UAP and MI, whereas levels of circulating let-7g-5p significantly discriminate UAP from MI. However, it is crucial to remember that conflicting findings from previous studies emphasize the need for further research to enable full comprehension of the potential function of miRNAs as diagnostic and/or prognostic biomarkers. Therefore, it is imperative to conduct more clinical validation studies to determine the clinical relevance of miRNAs and further functional studies to enlighten their role in the development of SAP, UAP, and MI.

## Supplementary Material

lmad094_suppl_Supplementary_Material

lmad094_suppl_Supplementary_File

## Abbreviations:

CAD: coronary artery disease
SAP: stable angina pectoris
UAP: unstable angina pectoris
MI: myocardial infarction
AMI: acute myocardial infarction
TC: total cholesterol
TG: triglycerides
CK-MB: creatine kinase–myocardial band
cTnI: cardiac troponin I
TyG: triglyceride-glucose
DAVID: Database for Annotation, Visualization, and Integrated Discovery
GO: gene ontology
KEGG: Kyoto Encyclopedia of Genes and Genomes
GAD: Genetic Association Database
T2DM: type 2 diabetes mellitus
ECs: endothelial cells
STEMI: ST-segment elevation myocardial infarction
NCCP: noncardiac chest pain
SIS: segment stenosis score
NSTEMI: non−ST-elevated MI
HUNT: Nord-Trøndelag Healthy
