# Supplementary material for: Alteration of circulating miRNAs during myocardial infarction and association with lipid levels
Source: Lab Med. 2023 Dec 4;55(3):361–72. doi: 10.1093/labmed/lmad094 (PMC11064099; doi:10.1093/labmed/lmad094)
Supplement: lmad094_suppl_Supplementary_Material [file lmad094_suppl_supplementary_material.docx]

**Alteration of circulating miRNAs during myocardial infarction and association with lipid levels**

**Supplemental Information**

**
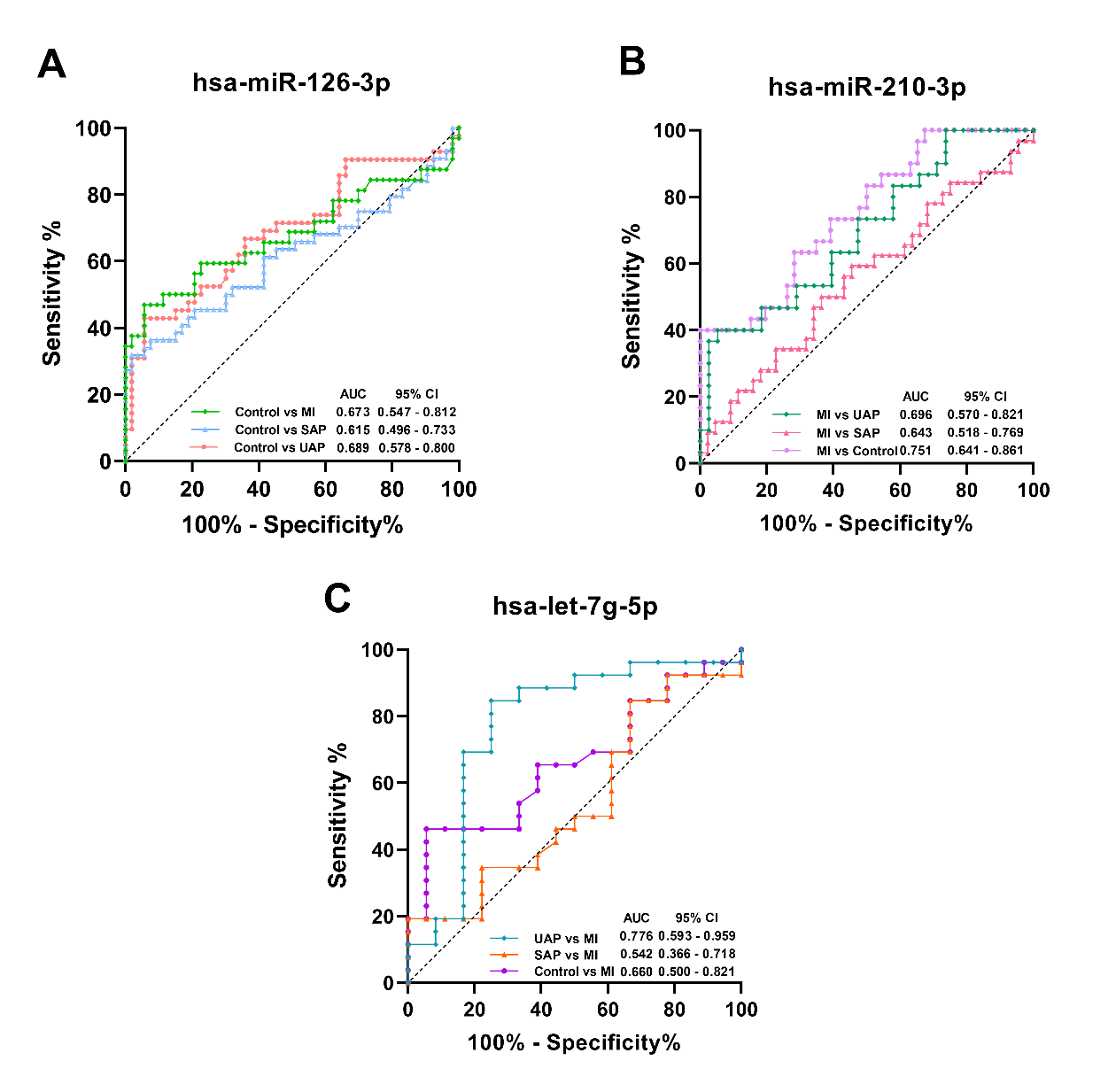
**

**Supp Figure 1. Sensitivity and specificity of circulating miR-126-3p, miR-210-3p, and let-7g-5p.** The receiver operating characteristic (ROC) curve analyses were performed for the determination of the discriminative ability of (A) miR-126-3p, (B) miR-210-3p, and (C) let-7g-5p among non-CAD, SAP, UAP, and MI.

**Supplemental Table**

**Table S1. Baseline characteristics of the study population**

| **Characteristics** | **non-CAD** (n=55) | **SAP** (n=48) | **UAP** (n=46) | **MI** (n=36) | ***p-value*** |
| --- | --- | --- | --- | --- | --- |
|  | mean ± SD; % | mean ± SD; % | mean ± SD; % | mean ± SD; % |  |
| Age (years) | 65.35 ± 8.89 | 52.04 ± 5.65 | 62.20 ± 12.83 | 54.03 ± 8.45 | ***<0.001*** |
| BMI (kg/m^2^) | 28.96 ± 4.25 | 28.68 ± 4.42 | 29.87 ± 3.96 | 28.91 ± 5.78 | *0.613* |
| TC (mg/dL) | 193.13 ± 36.58 | 220.48 ± 54.72 | 196.66 ± 45.98 | 183.58 ± 47.88 | ***0.002*** |
| HDL-C (mg/dL)^1^ | 42.0 (37.0-48.3) | 39.0 (33.4-46.0) | 38.5 (32.0-45.9) | 34.5 (28.6-44.8) | ***0.016*** |
| LDL-C(mg/dL) | 112.76 ± 31.67 | 129.32 ± 42.68 | 111.61 ± 35.38 | 110.33 ± 36.20 | ***0.046*** |
| TC/HDL-C ratio | 4.62 ± 1.30 | 5.66 ± 1.67 | 5.30 ± 1.65 | 5.29 ± 1.72 | ***0.010*** |
| LDL-C/HDL-C ratio | 2.71 ± 0.93 | 3.33 ± 1.25 | 2.98 ± 1.02 | 3.18 ± 1.24 | ***0.036*** |
| log10 (Triglyceride /HDL-C ratio) | 0.49 ± 0.27 | 0.67 ± 0.29 | 0.60 ± 0.33 | 0.66 ± 0.29 | ***0.013*** |
| Fasting triglyceride (mg/dL)^1^ | 134.0 (88.5-186.0) | 175.0 (129.0-261.0) | 159.0 (88.5-218.8) | 164.0 (115.3-218.8) | ***0.048*** |
| HbA1c (%)^1^ | 5.82 (5.22-6.22) | 5.86 (5.21-6.98) | 5.95 (5.43-7.01) | 6.18 (5.46-7.33) | *0.273* |
| Fasting glucose (mg/dL)^1^ | 98.0 (91.0-117.0) | 97.0 (90.3-112.5) | 104.0 (94.5-128.3) | 117.0 (95.5-145.0) | ***0.015*** |
| Stenosis (%) | 10.73 ± 12.00 | 87.81 ± 10.22 | 48.33 ± 36.85 | 77.14 ± 30.27 | ***<0.001*** |
| SYNTAX Score | - | 18.95 ± 12.96 | 7.59 ± 11.48 | 13.03 ± 12.01 | ***<0.001*** |
| Gensini Score | - | 63.57 ± 55.87 | 27.09 ± 42.34 | 48.97 ± 41.56 | ***<0.001*** |
| Type 2 diabetes mellitus^2^ | 30.9 (17) | 31.3 (15) | 37.0 (17) | 55.6 (20) | *0.077* |
| Obesity^2^ | 36.4 (20) | 29.8 (14) | 43.5 (20) | 41.7 (15) | *0.534* |
| Hypertension^2^ | 49.1 (27) | 54.2 (26) | 65.2 (30) | 44.4 (16) | *0.244* |
| CAD Family History^2^ | 32.7 (18) | 56.3 (27) | 37.0 (17) | 13.9 (5) | ***0.001*** |
| Current smokers^2^ | 36.4 (20) | 35.4 (17) | 30.4 (14) | 47.2 (17) | *0.470* |
| Lipid-lowering drug usage^2^ | 30.9 (17) | 35.4 (17) | 41.3 (19) | 30.6 (11) | *0.678* |
| Usage of antidiabetic drugs^2^ | 21.8 (12) | 31.3 (15) | 21.7 (10) | 33.3 (12) | *0.462* |
| Sex, Male^2^ | 76.4 (42) | 64.6 (31) | 56.5 (26) | 72.2 (26) | *0.168* |
|  | **non-CAD** (n=27) | **SAP** (n=31) | **UAP** (n=38) | **MI** (n=35) |  |
| cTnI (ng/L) | 4.40 (1.40-27.8) | 3.40 (1.60-23.6) | 5.05 (1.95-26.48) | 240.1 (37.5721.2) | ***<0.001*** |
| CK-MB (µg/L) | 1.00 (0.55-2.05) | 1.20 (0.80-1.60) | 1.20 (0.55-2.15) | 2.70 (1.30-14.9) | ***0.001*** |

^1^Nonparametric tests were performed, and results were given as median (25-75th percentile). ^2^Categorical variables expressed in percentages. BMI: Body mass index; CAD: Coronary artery disease; CK-MB: Creatine Kinase MB; cTnI: cardiac Troponin I; HbA1c: Glycated hemoglobin; HDL-C: High-density lipoprotein cholesterol; LDL-C: Low-density lipoprotein cholesterol; MI: Myocardial infarction; n: Number of individuals; SAP: Stable angina pectoris; TC: Total cholesterol; UAP: Unstable angina pectoris.
