# Supplementary material for: Alteration of circulating miRNAs during myocardial infarction and association with lipid levels
Source: Lab Med. 2023 Dec 4;55(3):361–72. doi: 10.1093/labmed/lmad094 (PMC11064099; doi:10.1093/labmed/lmad094)
Supplement: lmad094_suppl_Supplementary_File [file lmad094_suppl_supplementary_file.docx]

**Circulating miRNAs are associated with lipid levels and altered during myocardial infarction**

### Targets of miR-210-3p related with the cardiovascular diseases

##### miR-210-3p

PREDICTED

Intersection of ≥2 miRNA databases Intersection of 3 miRNA databases

BDKRB2, MEF2C, PPARA, THBS2

MALACARD

*ADIPOQ, ADM, AHSG, AKT1, BDKRB2, CASP3, CCR5, CIITA, CXCL12, DCN, F5, F7, F8, FGA, HFE, LRP6, LTA, MAPK8, MEF2A, MEF2C, MPO, NR3C2, PALLD, PAPPA, PDE4D, PPARA, PTPRC, STAT3, THBS2, THY1, TNFRSF1A, TNFSF4, VEGFA*

ABCA13, ABCC1, ABCC6, ABCG1, ABCG4, ABI2, ABLIM2, ACADSB, ACOT11, ACSL3, ADAM17, ADAM28, ADAMTS12, ADAMTS5, ADAMTS6, ADAMTSL5, ADARB1, ADCY5, ADCY9, ADCYAP1R1, ADIPOQ, ADM, ADORA2A, ADORA3, AFF2, AFTPH, AGPAT5, AGPS, AGTRAP, AHRR, AHSG, AKAP13, AKAP7, AKT1, ANKRD11, ANKS1A, ANO4, APOL1, AQP2, ARHGAP29, ARHGAP42, ARHGEF10, ARHGEF12, ARHGEF17, ARNT2, ARNTL, ARRB1, ARRDC4, ASAH2, ASB1, ASB6, ASCL1, ASTN2, ATF6, ATM, ATN1, ATP2B4, ATXN7L3B, AUTS2, B3GALT2, BACE1, BACH2, BARD1 BAZ2B, BBX, BDKRB2, BDNF, BMP6, BNC2, BTRC, C10orf55, C11orf87, C2, C2CD2, C3, C5orf38, CA12, CACNA1D, CACNA2D4, CADM1, CALCOCO1, CALM1, CAMK1D, CAMTA1, CAND1, CANT1, CAPZB, CBR4, CBX8, CCDC141, CCDC50, CCNG2, CCNJL, CCR5, CD247, CD93, CDC42BPA, CDC6, CDCA7, CDH13, CDH7, CDK14, CDKAL1, CDKN1A, CELF2, CELF4, CENPP, CHD2, CHEK2, CHRM2, CHRM3, CHRNA4, CHST9, CIITA, CLEC16A, CLSTN2, CMKLR1, CNKSR3, CNTN4, CNTNAP5, COBL, COL4A2, COL9A1, COQ6, CORO1C, CORO2B, COX10, CPEB2, CRBN, CREM, CRHBP, CSNK1E, CTNND2, CUBN, CUL3, CXCL12, CYLD, CYP1A1, CYP2S1, CYP3A5, DAB1, DAPK1, DCAF5, DCAF8L1, DCHS1, DCHS2, DCLK1, DCN, DDAH1, DDHD1, DDR2, DDX56, DNAJC11, DNAJC6, DPYSL2, DRD2, DYM, DYRK2, E2F3, E2F4, EGFLAM, EIF2AK1, ELF5, ELMO1, EPB41, EPHB3, ERAP1, ERC2, ERCC6, ERGIC1, ERMN, EVL, EYA1, F5, F7, F8, FADS1, FAM107B, FAM13A, FAM167A, FAM189A1, FAM84A, FBLN5, FBXL7, FEZ2, FGA, FGF1, FGF14, FGFR1, FHL5, FIGN, FKBP1A, FKBP1B, FLRT2, FMN1, FOXO3, FOXP1, FRMD6, FRMPD4, FTO, FUT3, GABRG3, GABRP, GALNT2, GALR1, GAS7, GHR, GIMAP1, GINS3, GLI1, GLIS1, GNA15, GNAS, GNB1L, GOLPH3, GPR101, GPR37L1, GPRIN3, GRAMD1B, GRID1, GRIN2A, GUCY1A2, HDAC4, HDLBP, HDX, HFE, HHIPL1, HIF1AN, HIPK3, HIVEP3, HK2, HLA-DPB1, HMGA2, HMP19, HNF1A, HNF4A, HPCAL1, HUS1, IER5, IGF1R, IGF2, IGSF5, IKZF2, IL12RB2, IL18BP, IL18R1, IL2RA, ING3, INHBA, INHBB, INO80D, INPP5D, INSIG1, IP6K3, IPO5, IQSEC1, IQSEC3, IRAK1, IRAK4, ITPK1, JMJD1C, JUP, KCNE1L, KCNJ6, KCNK12, KCNMA1, KCNN3, KCNS3, KCTD12, KCTD7, KIAA0040, KIAA0232, KIAA1549, KIAA1958, KIF13B, KIF1A, KLB, KLF12, KLF7, KLHDC4, KLHL21, KLHL31, LAX1, LEPR, LGSN, LHFP, LHX5, LIMK1, LIPE, LOXL3, LPCAT4, LPIN1, LPP, LRIG1, LRP6, LRPAP1, LRRC20, LSM11, LTA, LZTS1, MAFB, MAGI2, MAML3, MAP4, MAPK1, MAPT, MBP, MDH1B, MDM2, MEF2A, MEF2C, MEF2D, MGAT5, MGLL, MGST2, MICALCL, MID1, MPO, MPPED2, MRAS, MREG, MSI2, MTUS1, MYH11, MYH9, MYLK, MYO16, MYO5B, MYO9B, MYOCD, MYT1L, NAV2, NCALD, NCAM1, NDRG4, NFATC2, NKAIN2, NLRP3, NMNAT2, NMNAT3, NNT, NPNT, NPR3, NR3C1, NR3C2, NRAS, NRG1, NRN1, NTRK2, NUP210, NVL, ODF2L, ONECUT2, OPCML, OPHN1, OSBPL3, OXTR, P2RX6, P2RY2, P4HA2, PACS2, PADI3, PAFAH1B1, PAFAH2, PALLD, PANK3, PAPPA, PARK2, PAX3, PAX5, PAX6, PBX1, PDCD2, PDCD6IP, PDE10A, PDE1C, PDE3A, PDE4D, PDE8B, PDZD2, PERP, PGPEP1, PHACTR2, PHEX, PIGG, PIK3C3, PKDCC, PLEKHG1, PLSCR4, PLXDC2, PLXNA4, POGK, POLR1D, PPARA, PPARGC1A, PPM1H, PPP1R3B, PPP1R7, PPP1R9A, PPP2R2C, PPP2R5E, PRC1, PRDM15, PRDM8, PRICKLE1, PRIMA1, PRKAA2, PRKAG2, PRKCA, PRLR, PROK1, PRRC1, PRRX1, PRUNE2, PTCHD1, PTPRC, PTPRK, QKI, RAB11FIP4, RAB6B, RABGAP1L, RAI14, RALYL, RANBP17, RASGEF1A, RASGRF2, RBBP4, RBFOX1, RBM33, RBM38, REEP3, RGMA, RGS17, RGS4, RGS5, RGS7, RGS8, RHOJ, RIMS1, RIMS2, RNF111, RNF130, RNF24, RNLS, ROBO2, ROR1, ROR2, RORA, RPA1, RPTOR, RXRA, RYBP, SAMD12, SCARA3, SCCPDH, SCD5, SCG5, SCN1B, SCN4B, SCNN1A, SDHAF2, SEMA3A, SEMA5A, SEMA6A, SESN3, SETBP1, SH2B3, SHANK2, SHB, SHH, SIN3A, SIPA1L3, SLAMF6, SLC10A7, SLC12A3, SLC14A1, SLC19A1, SLC1A2, SLC20A1, SLC22A3, SLC23A2, SLC24A2, SLC25A24, SLC26A9, SLC38A7, SLC40A1, SLC44A1, SLC44A5, SLC5A12, SLC5A3, SLC6A2, SLC6A9, SLC7A11, SLC7A8, SLC8A1, SLC9A6, SLCO3A1, SLCO5A1, SLITRK3, SMAD2, SMAD4, SMAD5, SMARCA4, SMPD1, SNN, SOD2, SORBS1, SORCS1, SOX11, SPAG6, SPC24, SPTBN4, SRD5A1, SRGAP1, SS18L1, ST6GAL2, ST8SIA5, STAT3, STAT6, STK35, STRN, STX18, SUMF1, SUSD4, SV2B, SVOP, SYK, SYNJ2, SYNPO2, SYT6, SYT9, SYTL4, TACSTD2, TANC2, TAZ, TBC1D1, TBC1D16, TBC1D5, TBCD, TBL3, TBX3, TBXAS1, TCTN3, TET2, THAP6, THBS2, THOC5, THPO, THSD4, THSD7A, THY1, TIGIT, TMEM132D, TMEM163, TMOD2, TMPRSS4, TMTC1, TNFRSF1A, TNFSF4, TNFSF9, TNRC6B, TNS1, TOR1A, TP73, TPM1, TRAF1, TRDMT1, TRIB1, TRIM33, TRIM71, TRPC4, TRPC5, TRPS1, TSC1, TSHR, TSPAN9, TTLL11, TTLL6, UBE2E2, UBE2Z, UBE4B, UBTD2, UCP3, UNG, USP3, USP6NL, VAT1L, VEGFA, VIPR2, VTCN1, VWA2, WASF2, WASF3, WDR12, WDR37, WDR49, WDR7, WIPI2, WWC2, WWP2, XCL1, YES1, YPEL2, ZMYND11, ZNF264, ZNF516, ZNF608, ZNF618, ZNF778, ZNF827, ZNF831

GAD

ABI2, ADARB1, ADCY5, AFF2, ANO4, ARRDC4, ASAH2, ATN1, BARD1, BDKRB2, BDNF, BMP6, C2CD2, C5orf38, CA12, CAMTA1, CELF2, CHRM3, CLEC16A, CNTNAP5, COL4A2, CORO2B, CYP1A1, DCAF5, DNAJC6, DYRK2, E2F3, EPB41, EYA1, FAM13A, GINS3, HDLBP, HIVEP3, HK2, IER5, IGF2, INHBB, IP6K3, IQSEC1, IQSEC3, KIAA1958, KIF13B, KLF12, KLF7, KLHDC4, LGSN, LIMK1, LRIG1, MEF2C, MYH11, MYO16, NMNAT2, PARK2, PAX5, PAX6, PBX1, PDE1C, PHEX, PPARA, PRLR, QKI, RGS7, SAMD12, SCARA3, SH2B3, SIN3A, SLC1A2, SLC22A3, SLC24A2, SLC38A7, SLC5A12, SMAD4, SMARCA4, SORBS1, ST6GAL2, STAT6, STK35, SV2B, TBC1D16, THBS2, TNRC6B, TNS1, TP73, UCP3, USP6NL, WDR7, ZNF618,

**GO Biological Process Terms (miR-210-3p)**

| **Term** | **Overlap P-value** | | **Adjusted P-value** |
| --- | --- | --- | --- |
| regulation of transcription, DNA-templated (GO:0006355) | 338/2244 | *6,56E-10* | *1,70E-06* |
| regulation of transcription by RNA polymerase II (GO:0006357) | 333/2206 | *7,02E-10* | *1,70E-06* |
| positive regulation of transcription, DNA-templated (GO:0045893) | 198/1183 | *1,21E-09* | *1,95E-06* |
| positive regulation of transcription by RNA polymerase II (GO:0045944) | 156/908 | *1,32E-08* | *1,60E-05* |
| axon guidance (GO:0007411) | 50/203 | *3,91E-08* | *3,79E-05* |
| cellular protein modification process (GO:0006464) | 164/1025 | *6,87E-07* | *5,54E-04* |
| axonogenesis (GO:0007409) | 52/240 | *1,58E-06* | *1,09E-03* |
| phosphorylation (GO:0016310) | 76/400 | *1,83E-06* | *1,11E-03* |
| negative regulation of transcription, DNA-templated (GO:0045892) | 151/948 | *2,49E-06* | *1,28E-03* |
| vascular transport (GO:0010232) | 25/84 | *2,64E-06* | *1,28E-03* |
| negative regulation of transcription by RNA polymerase II (GO:0000122) | 115/684 | *3,24E-06* | *1,43E-03* |
| regulation of cellular protein localization (GO:1903827) | 17/46 | *4,09E-06* | *1,57E-03* |
| transport across blood-brain barrier (GO:0150104) | 25/86 | *4,22E-06* | *1,57E-03* |
| protein phosphorylation (GO:0006468) | 87/496 | *9,83E-06* | *3,35E-03* |
| positive regulation of nucleic acid-templated transcription (GO:1903508) | 89/511 | *1,04E-05* | *3,35E-03* |
| amino acid import across plasma membrane (GO:0089718) | 11/24 | *1,93E-05* | *5,56E-03* |
| ion transport (GO:0006811) | 29/116 | *1,95E-05* | *5,56E-03* |
| protein autophosphorylation (GO:0046777) | 35/159 | *5,38E-05* | *1,45E-02* |
| negative regulation of intracellular signal transduction (GO:1902532) | 41/198 | *5,80E-05* | *1,48E-02* |
| positive regulation of cell projection organization (GO:0031346) | 28/117 | *6,27E-05* | *1,52E-02* |
| regulation of gene expression (GO:0010468) | 160/1079 | *6,91E-05* | *1,59E-02* |
| cation transport (GO:0006812) | 37/174 | *7,29E-05* | *1,60E-02* |
| import into cell (GO:0098657) | 14/41 | *7,94E-05* | *1,63E-02* |
| regulation of axonogenesis (GO:0050770) | 16/51 | *8,06E-05* | *1,63E-02* |
| protein localization to membrane (GO:0072657) | 40/195 | *8,82E-05* | *1,71E-02* |
| nervous system development (GO:0007399) | 76/447 | *1,00E-04* | *1,85E-02* |
| transmembrane receptor protein tyrosine kinase signaling pathway (GO:0007169) | 70/404 | *1,03E-04* | *1,85E-02* |
| chemical synaptic transmission (GO:0007268) | 56/306 | *1,14E-04* | *1,98E-02* |
| protein localization to cell periphery (GO:1990778) | 31/140 | *1,25E-04* | *2,02E-02* |
| cardiac muscle cell differentiation (GO:0055007) | 10/24 | *1,25E-04* | *2,02E-02* |
| positive regulation of axonogenesis (GO:0050772) | 14/43 | *1,43E-04* | *2,23E-02* |
| protein localization to plasma membrane (GO:0072659) | 30/136 | *1,71E-04* | *2,58E-02* |
| neuron development (GO:0048666) | 31/143 | *1,88E-04* | *2,75E-02* |
| cellular response to peptide hormone stimulus (GO:0071375) | 25/106 | *1,94E-04* | *2,76E-02* |
| regulation of lipid biosynthetic process (GO:0046890) | 12/35 | *2,44E-04* | *3,26E-02* |
| regulation of muscle cell differentiation (GO:0051147) | 12/35 | *2,44E-04* | *3,26E-02* |
| positive regulation of signal transduction (GO:0009967) | 47/252 | *2,49E-04* | *3,26E-02* |
| negative regulation of protein kinase activity (GO:0006469) | 22/90 | *2,67E-04* | *3,33E-02* |
| regulation of vesicle-mediated transport (GO:0060627) | 23/96 | *2,69E-04* | *3,33E-02* |
| inorganic cation transmembrane transport (GO:0098662) | 50/274 | *2,77E-04* | *3,36E-02* |
| modulation of chemical synaptic transmission (GO:0050804) | 25/109 | *3,07E-04* | *3,62E-02* |
| limb morphogenesis (GO:0035108) | 9/22 | *3,24E-04* | *3,73E-02* |

**GO Molecular Function Terms (miR-210-3p)**

| **Term** | **Overlap P-value** | | **Adjusted P-value** |
| --- | --- | --- | --- |
| sequence-specific double-stranded DNA binding (GO:1990837) | 125/712 | *1,13E-07* | *8,29E-05* |
| RNA polymerase II transcription regulatory region sequence-specific DNA binding (GO:0000977) | 211/1359 | *1,69E-07* | *8,29E-05* |
| RNA polymerase II cis-regulatory region sequence-specific DNA binding (GO:0000978) | 181/1149 | *5,15E-07* | *1,68E-04* |
| cis-regulatory region sequence-specific DNA binding (GO:0000987) | 180/1149 | *8,03E-07* | *1,97E-04* |
| sequence-specific DNA binding (GO:0043565) | 115/707 | *1,54E-05* | *3,02E-03* |
| double-stranded DNA binding (GO:0003690) | 106/651 | *3,15E-05* | *5,15E-03* |
| DNA-binding transcription factor binding (GO:0140297) | 43/208 | *4,04E-05* | *5,66E-03* |
| transcription cis-regulatory region binding (GO:0000976) | 91/549 | *5,78E-05* | *7,08E-03* |
| SH3 domain binding (GO:0017124) | 18/62 | *9,23E-05* | *1,00E-02* |
| kinase binding (GO:0019900) | 77/461 | *1,63E-04* | *1,59E-02* |
| amino acid transmembrane transporter activity (GO:0015171) | 15/49 | *1,82E-04* | *1,62E-02* |
| protein serine/threonine kinase activity (GO:0004674) | 60/344 | *2,60E-04* | *2,12E-02* |
| branched-chain amino acid transmembrane transporter activity (GO:0015658) | 5/7 | *2,90E-04* | *2,19E-02* |
| carboxylic acid transmembrane transporter activity (GO:0046943) | 16/57 | *3,41E-04* | *2,36E-02* |
| DNA-binding transcription repressor activity, RNA polymerase II-specific (GO:0001227) | 47/256 | *3,61E-04* | *2,36E-02* |
| protein kinase binding (GO:0019901) | 81/506 | *4,46E-04* | *2,73E-02* |
| organic anion transmembrane transporter activity (GO:0008514) | 30/144 | *4,84E-04* | *2,79E-02* |
| transcription regulatory region nucleic acid binding (GO:0001067) | 40/212 | *5,46E-04* | *2,97E-02* |
| myosin V binding (GO:0031489) | 7/15 | *5,89E-04* | *3,04E-02* |
| kinesin binding (GO:0019894) | 10/29 | *7,61E-04* | *3,73E-02* |
| dynactin binding (GO:0034452) | 6/12 | *9,48E-04* | *4,33E-02* |
| DNA-binding transcription activator activity, RNA polymerase II-specific (GO:0001228) | 56/333 | *1,01E-03* | *4,33E-02* |
| Hsp90 protein binding (GO:0051879) | 11/35 | *1,02E-03* | *4,33E-02* |

**Top 20 KEGG Terms for the intersected targets of miR-210-3p**

**p-value**

**(Corrected with % of**

**Term**

**p-value**

**Benjamini-**

**Hochberg)**

**Associated Number**

**ID**

**Genes**

**of Genes Associated Genes Found**

KEGG:04360 **Axon guidance** *7,90E-10 1,85E-07* 28,57 52

**Oxytocin signaling**

KEGG:04921 **pathway** *3,55E-06 2,77E-04* 25,32 39

*ABL1, ABLIM2, ARHGEF12, BOC, CAMK2A, CAMK2B, CDC42, CXCL12, DPYSL2, EFNA1, EFNA3, EFNB1, ENAH, EPHA2, EPHB3, FZD3, GDF7, GSK3B, LIMK1, MAPK1, MYL9, NFATC2, NFATC3, NRAS, NTN1, NTNG2, PAK2, PARD6G, PDPK1, PLXNA1, PLXNA4, PRKCA, PRKCZ, RGMA, ROBO2, ROCK1, SEMA3A, SEMA3G, SEMA4F, SEMA5A, SEMA5B, SEMA6A, SHH, SRGAP1, SSH1, SSH2, SSH3, TRPC3, TRPC4, TRPC5, UNC5A, UNC5D*

*ADCY1, ADCY5, ADCY7, ADCY9, CACNA1D, CACNA2D4, CACNB4, CACNG7, CACNG8, CALM1, CALML4, CAMK1D, CAMK2A, CAMK2B, CDKN1A, ELK1, GNAS, GUCY1A2, JMJD7-PLA2G4B, KCNJ6, MAPK1, MEF2C, MYL9, MYLK, NFATC2, NFATC3, NRAS, OXTR, PIK3R5, PLCB1, PPP1CB, PPP1R12A, PPP1R12C, PRKAA2, PRKACB, PRKAG2, PRKCA, RCAN1, ROCK1*

KEGG:04740 **Olfactory transduction** *2,53E-06 2,96E-04* 5,42 24

**Adrenergic signaling**

KEGG:04261 **in cardiomyocytes** *3,24E-05 1,52E-03* 24,00 36

*ANO2, ARRB1, CALM1, CALML4, CAMK2A, CAMK2B, CNGA2, GNG7, NCALD, OR10H1, OR1D2, OR2G6, OR2L2, OR2T6, OR51E2, OR51L1, OR52N1, OR56B1, OR6C75, PDE1B, PDE1C, PRKACB, SLC8A1, SLC8A2 ADCY1, ADCY5, ADCY7, ADCY9, AKT1, ATP2B3, ATP2B4, CACNA1D, CACNA2D4, CACNB4, CACNG7, CACNG8, CALM1, CALML4, CAMK2A, CAMK2B, CREB1, CREB3L3, CREM, GNAS, MAPK1, PIK3R5, PLCB1, PPP1CB, PPP2R2C, PPP2R3B, PPP2R5D, PPP2R5E, PRKACB, PRKCA, SCN1B, SCN4B, SLC8A1, SLC8A2, TPM1, TPM3*

**cGMP-PKG signaling**

KEGG:04022 **pathway** *2,94E-05 1,72E-03* 23,35 39

*ADCY1, ADCY5, ADCY7, ADCY9, ADORA3, AKT1, ATP2B3, ATP2B4, BDKRB2, CACNA1D, CALM1, CALML4, CREB1, CREB3L3, GNA11, GTF2IRD1, GUCY1A2, KCNMA1, MAPK1, MEF2A, MEF2C, MEF2D, MYL9, MYLK, NFATC2, NFATC3, PDE3A, PIK3R5, PLCB1, PPIF, PPP1CB, PPP1R12A, ROCK1, SLC25A4, SLC25A6, SLC8A1, SLC8A2, SRF, VASP*

**Dopaminergic**

KEGG:04728 **synapse** *6,48E-05 2,53E-03* 24,24 32

*[ADCY5, AKT1, ARNTL, ARRB1, CACNA1A, CACNA1D, CALM1, CALML4, CAMK2A, CAMK2B, CREB1, CREB3L3, DRD2, DRD5, GNAS, GNB5, GNG2, GNG7, GRIN2A, GSK3B, KCNJ6, KIF5A, MAPK10, MAPK8, PLCB1, PPP1CB, PPP2R2C, PPP2R3B, PPP2R5D, PPP2R5E, PRKACB, PRKCA]*

KEGG:05032 **Morphine addiction** *1,26E-04 4,21E-03* 26,37 24

**cAMP signaling**

KEGG:04024 **pathway** *1,64E-04 4,26E-03* 20,83 45

KEGG:04140 **Autophagy** *1,51E-04 4,43E-03* 23,36 32

*[ADCY1, ADCY5, ADCY7, ADCY9, ARRB1, CACNA1A, GABRB3, GABRG2, GABRG3, GABRP, GABRQ, GNAS, GNB5, GNG2, GNG7, KCNJ6, PDE10A, PDE1B, PDE1C, PDE3A, PDE4D, PDE8B, PRKACB, PRKCA]*

*ACOX1, ADCY1, ADCY5, ADCY7, ADCY9, ADCYAP1R1, ADORA2A, AKT1, ATP2B3, ATP2B4, BDNF, CACNA1D, CALM1, CALML4, CAMK2A, CAMK2B, CHRM2, CNGA2, CREB1, CREB3L3, DRD2, DRD5, GLI1, GNAS, GRIN1, GRIN2A, GRIN3B, HCAR1, HCN4, LIPE, MAPK1, MAPK10, MAPK8, MYL9, OXTR, PDE10A, PDE3A, PDE4D, PPARA, PPP1CB, PPP1R12A, PRKACB, ROCK1, TSHR, VIPR2*

*AKT1, ATG4A, ATG7, ATG9A, CFLAR, CTSB, DAPK1, DEPTOR, ERN1, IGF1R, MAPK1, MAPK10, MAPK8, MRAS, MTMR3, NRAS, NRBF2, PDPK1, PIK3C3, PRKAA2, PRKACB, PTEN, RAB33B, RAB7A, RAB7B, RAB8A, RPTOR, SH3GLB1, TSC1, VMP1, WIPI2, ZFYVE1*

KEGG:04144 **Endocytosis** *2,33E-04 5,44E-03* 19,84 50

KEGG:04725 **Cholinergic synapse** *3,42E-04 7,28E-03* 23,89 27

**Apelin signaling**

KEGG:04371 **pathway** *4,32E-04 7,77E-03* 22,63 31

KEGG:04014 **Ras signaling pathway** *4,30E-04 8,38E-03* 19,83 46

**AMPK signaling**

KEGG:04152 **pathway** *5,08E-04 8,49E-03* 23,33 28

**Hedgehog signaling**

KEGG:04340 **pathway** *6,03E-04 9,41E-03* 28,57 16

**Insulin signaling**

KEGG:04910 **pathway** *7,82E-04 1,14E-02* 21,90 30

*AP2A2, ARFGEF1, ARRB1, ASAP1, CAPZB, CCR5, CDC42, CHMP4B, CLTB, CYTH1, CYTH2, DNAJC6, EHD2, EHD3, EPN1, EPN2, GIT2, GRK1, IGF1R, IL2RA, IQSEC1, IQSEC3, KIF5A, LDLRAP1, MDM2, MVB12B, PARD6G, PDCD6IP, PRKCZ, PSD3, RAB11B, RAB11FIP4, RAB35, RAB5B, RAB7A, RAB8A, RABEP1, RNF41, RUFY2, SH3GLB1, SH3GLB2, SMAD2, STAM, STAMBP, TFRC, VPS37D, VPS45, WIPF1, WIPF2, ZFYVE16*

*ADCY1, ADCY5, ADCY7, ADCY9, AKT1, CACNA1A, CACNA1D, CAMK2A, CAMK2B, CHRM2, CHRM3, CHRNA4, CHRNA7, CREB1, CREB3L3, GNA11, GNB5, GNG2, GNG7, KCNJ6, KCNQ4, MAPK1, NRAS, PIK3R5, PLCB1, PRKACB, PRKCA*

*[ADCY1, ADCY5, ADCY7, ADCY9, AKT1, CALM1, CALML4, GNB5, GNG2, GNG7, HDAC4, LIPE, MAPK1, MEF2A, MEF2C, MEF2D, MRAS, MYLK, NRAS, PIK3C3, PIK3R5, PLCB1, PLIN1, PPARGC1A, PRKAA2, PRKACB, PRKAG2, SLC8A1, SLC8A2, SMAD2, SMAD4]*

*[ABL1, ABL2, AKT1, BDNF, CALM1, CALML4, CDC42, EFNA1, EFNA3, ELK1, EPHA2, ETS1, FGF1, FGFR1, FLT4, GAB1, GNB5, GNG2, GNG7, GRIN1, GRIN2A, IGF1R, IGF2, IKBKB, JMJD7-PLA2G4B, KSR1, MAPK1, MAPK10, MAPK8, MRAS, NGFR, NRAS, NTRK2, PAK2, PLA2G2F, PRKACB, PRKCA, RAB5B, RAPGEF5, RASAL2, RASGRF2, RASSF5, REL, STK4, SYNGAP1, VEGFA]*

*ADIPOQ, AKT1, CREB1, CREB3L3, FASN, FOXO3, G6PC, HNF4A, IGF1R, LEPR, LIPE, MLYCD, PDPK1, PFKFB3, PPARGC1A, PPP2R2C, PPP2R3B, PPP2R5D, PPP2R5E, PRKAA2, PRKAG2, RAB11B, RAB8A, RPTOR, SCD5, STRADB, TBC1D1, TSC1*

*ARRB1, BOC, BTRC, CDON, CSNK1E, CUL3, GLI1, GSK3B, IQCE, KIF3A, KIF7, MGRN1, PRKACB, SHH, SPOPL, SUFU*

*AKT1, CALM1, CALML4, CRKL, ELK1, FASN, G6PC, GSK3B, HK2, IKBKB, INPP5A, LIPE, MAPK1, MAPK10, MAPK8, NRAS, PDPK1, PPARGC1A, PPP1CB, PPP1R3B, PPP1R3E, PPP1R3F, PRKAA2, PRKACB, PRKAG2, PRKAR2A, PRKCZ, RPTOR, SORBS1, TSC1*

**Neurotrophin**

KEGG:04722 **signaling pathway** *8,60E-04 1,18E-02* 22,69 27

**Signaling pathways regulating pluripotency of stem**

KEGG:04550 **cells** *9,51E-04 1,24E-02* 21,68 31

*ABL1, AKT1, BDNF, CALM1, CALML4, CAMK2A, CAMK2B, CDC42, CRKL, FOXO3, GAB1, GSK3B, IKBKB, IRAK1, IRAK3, IRAK4, MAP2K7, MAPK1, MAPK10, MAPK8, NGFR, NRAS, NTRK2, PDPK1, PSEN1, SH2B3, TP73*

*ACVR1B, ACVR1C, AKT1, APC2, DVL1, DVL3, FGFR1, FZD1, FZD3, GSK3B, HOXA1, IGF1R, INHBA, INHBB, JARID2, KAT6A, LHX5, LIF, MAPK1, NRAS, OTX1, PAX6, PCGF3, POU5F1B, RIF1, SKIL, SMAD2, SMAD4, SMAD5, STAT3, TBX3*

**Vascular smooth**

KEGG:04270 **muscle contraction** *1,06E-03 1,30E-02* 21,80 29

**Rap1 signaling**

KEGG:04015 **pathway** *1,16E-03 1,36E-02* 19,52 41

*ADCY1, ADCY5, ADCY7, ADCY9, ADM, ADORA2A, ARHGEF12, CACNA1D, CALM1, CALML4, GNA11, GNAS, GUCY1A2, JMJD7-PLA2G4B, KCNMA1, MAPK1, MYH11, MYH9, MYL9, MYLK, PLA2G2F, PLCB1, PPP1CB, PPP1R12A, PPP1R12C, PRKACB, PRKCA, PTGIR, ROCK1*

*ADCY1, ADCY5, ADCY7, ADCY9, ADORA2A, AKT1, CALM1, CALML4, CDC42, CRKL, DRD2, EFNA1, EFNA3, ENAH, EPHA2, EVL, FARP2, FGF1, FGFR1, FLT4, GNAS, GRIN1, GRIN2A, IGF1R, MAGI2, MAGI3, MAPK1, MRAS, NGFR, NRAS, PARD6G, PFN2, PLCB1, PRKCA, PRKCZ, RAPGEF5, RASSF5, SIPA1L3, TLN2, VASP, VEGFA*

**Targets of miR-126-3p related with the cardiovascular diseases**

#### miR-126-3p

PREDICTED

Intersection of ≥2 miRNA databases Intersection of 3 miRNA databases

*APOA5, LRP6, VCAM1 LRP6, VCAM1*

MALACARD

*ADAM9, ANKS1A, APOA5, ARAP2, BACE1, BCL2, CENPP, CHST3, DIP2C, EGFR, FBN2, FBXO33, FERMT1, FOXO3, FRS2, GOLPH3, GRIN2B, HERPUD1, IRS1, IRS2, ITGA6, KCNJ1, KIF1A, LNPEP, LRP6, NF1, PAG1, PBLD, PNPT1, PRKCA, PTCH1, RGS3, RNF182, RNF4, SAMD12, SDC2, SLC19A2, SLC38A7, SLC4A4, SLC9A6, SMOC2, STX8, THAP6, TNFRSF10B, TRIM67, TRPS1, TSC1, VCAM1,*

GAD

*ARAP2, CHST3, DIP2C, FBXO33, GOLPH3, IRS1, ITGA6, LRP6, PTCH1, RGS3, RNF182, TNFRSF10B, TSC1, VCAM1*

*ZNF813*

**GO Biological Process Terms (miR-126-3p)**

| **Term** | **Overlap P-value** | | **Adjusted P-value** |
| --- | --- | --- | --- |
| positive regulation of cellular catabolic process (GO:0031331) | 10/141 | *1,71E-07* | *2,29E-04* |
| positive regulation of intracellular signal transduction (GO:1902533) | 18/546 | *3,05E-07* | *2,29E-04* |
| positive regulation of protein kinase B signaling (GO:0051897) | 9/161 | *5,19E-06* | *2,43E-03* |
| phosphatidylinositol 3-kinase signaling (GO:0014065) | 5/34 | *6,47E-06* | *2,43E-03* |
| transmembrane receptor protein tyrosine kinase signaling pathway (GO:0007169) | 13/404 | *1,89E-05* | *5,00E-03* |
| phosphatidylinositol-mediated signaling (GO:0048015) | 6/71 | *2,00E-05* | *5,00E-03* |
| axon guidance (GO:0007411) | 9/203 | *3,32E-05* | *7,12E-03* |
| regulation of protein kinase B signaling (GO:0051896) | 9/207 | *3,87E-05* | *7,26E-03* |
| positive regulation of macromolecule biosynthetic process (GO:0010557) | 7/129 | *7,21E-05* | *1,20E-02* |
| positive regulation of cellular biosynthetic process (GO:0031328) | 8/180 | *9,00E-05* | *1,35E-02* |
| embryonic appendage morphogenesis (GO:0035113) | 3/13 | *1,28E-04* | *1,75E-02* |
| response to cytokine (GO:0034097) | 7/150 | *1,85E-04* | *2,20E-02* |
| regulation of phosphatidylinositol 3-kinase signaling (GO:0014066) | 6/106 | *1,90E-04* | *2,20E-02* |
| positive regulation of cell migration (GO:0030335) | 9/269 | *2,82E-04* | *3,03E-02* |
| regulation of cell migration (GO:0030334) | 11/408 | *3,89E-04* | *3,89E-02* |
| regulation of anatomical structure morphogenesis (GO:0022603) | 6/123 | *4,24E-04* | *3,91E-02* |
| posttranscriptional regulation of gene expression (GO:0010608) | 4/46 | *4,61E-04* | *3,91E-02* |
| ephrin receptor signaling pathway (GO:0048013) | 5/82 | *4,69E-04* | *3,91E-02* |
| positive regulation of neuron death (GO:1901216) | 4/47 | *5,01E-04* | *3,96E-02* |
| rRNA transport (GO:0051029) | 2/5 | *6,03E-04* | *4,13E-02* |
| axonogenesis (GO:0007409) | 8/240 | *6,31E-04* | *4,13E-02* |
| positive regulation of lipid catabolic process (GO:0050996) | 3/22 | *6,55E-04* | *4,13E-02* |
| limb morphogenesis (GO:0035108) | 3/22 | *6,55E-04* | *4,13E-02* |
| inositol lipid-mediated signaling (GO:0048017) | 4/51 | *6,85E-04* | *4,13E-02* |
| regulation of intracellular signal transduction (GO:1902531) | 11/437 | *6,88E-04* | *4,13E-02* |
| positive regulation of neuron apoptotic process (GO:0043525) | 3/24 | *8,51E-04* | *4,87E-02* |
| negative regulation of MAPK cascade (GO:0043409) | 5/94 | *8,75E-04* | *4,87E-02* |

| **GO Molecular Function Terms (miR-126-3p)** |  | | |
| --- | --- | --- | --- |
| **Term** | **Overlap P-value** |  | **Adjusted P-value** |
| aromatic amino acid transmembrane transporter activity (GO:0015173) | 3/9 | *3,85E-05* | *1,06E-02* |

**Top 20 KEGG Terms for the intersected targets of miR-126-3p**

| **ID** | **Term** | **p-value** | **p-value (Corrected with Benjamini- Hochberg)** | **% of Associated Genes** | **Number of Genes** | **Associated Genes Found** |
| --- | --- | --- | --- | --- | --- | --- |
|  |  |  |  |  |  | *BCL2, CRK, FOXO3, FRS2,* |
|  | **Neurotrophin signaling** |  |  |  |  | *GAB1, IRS1, PIK3CD,* |
| KEGG:04722 | **pathway** | *9,27E-07* | *3,06E-05* | 7,56 | 9 | *PIK3R2, RPS6KA6* |
|  | **Aldosterone-regulated** |  |  |  |  | *IRS1, KCNJ1, PIK3CD,* |
| KEGG:04960 | **sodium reabsorption** | *1,69E-05* | *2,79E-04* | 13,51 | 5 | *PIK3R2, PRKCA* |
|  |  |  |  |  |  | *CRK, EGFR, GAB1, PIK3CD,* |
| KEGG:04012 | **ErbB signaling pathway** | *1,01E-04* | *8,32E-04* | 7,06 | 6 | *PIK3R2, PRKCA* |
|  |  |  |  |  |  | *EGFR, FOXO3, IRS1, IRS2,* |
| KEGG:04068 | **FoxO signaling pathway** | *1,52E-04* | *8,38E-04* | 5,34 | 7 | *PIK3CD, PIK3R2, PLK2* |
|  | **Longevity regulating** |  |  |  |  | *FOXO3, IRS1, IRS2, PIK3CD,* |
| KEGG:04211 | **pathway** | *1,30E-04* | *8,60E-04* | 6,74 | 6 | *PIK3R2, TSC1* |
|  | **Growth hormone** |  |  |  |  |  |
|  | **synthesis, secretion and** |  |  |  |  | *CRK, IRS1, IRS2, PIK3CD,* |
| KEGG:04935 | **action** | *8,32E-05* | *9,15E-04* | 5,88 | 7 | *PIK3R2, PRKCA, SSTR3* |
|  | **Longevity regulating** |  |  |  |  | *FOXO3, IRS1, IRS2, PIK3CD,* |
| KEGG:04213 | **pathway** | *2,11E-04* | *9,96E-04* | 8,06 | 5 | *PIK3R2* |
|  |  |  |  |  |  | *IRS1, PIK3CD, PIK3R2,* |
|  |  |  |  |  |  | *PRKCA, RPS6KA6, SLC7A5,* |
| KEGG:04150 | **mTOR signaling pathway** | *4,28E-04* | *1,57E-03* | 4,52 | 7 | *TSC1* |
|  |  |  |  |  |  | *EGFR, FOXO3, PIK3CD,* |
| KEGG:05223 | **Non-small cell lung cancer** | *4,27E-04* | *1,76E-03* | 6,94 | 5 | *PIK3R2, PRKCA* |
| KEGG:04930 | **Type II diabetes mellitus** | *7,09E-04* | *2,13E-03* | 8,70 | 4 | *IRS1, IRS2, PIK3CD, PIK3R2* |
|  |  |  |  |  |  | *FOXO3, IRS1, IRS2, PIK3CD,* |
| KEGG:04152 | **AMPK signaling pathway** | *6,60E-04* | *2,18E-03* | 5,00 | 6 | *PIK3R2, TSC1* |
|  |  |  |  |  |  | *BCL2, IRS1, IRS2, PIK3CD,* |
| KEGG:04140 | **Autophagy** | *1,32E-03* | *3,63E-03* | 4,38 | 6 | *PIK3R2, TSC1* |
|  |  |  |  |  |  | *CRK, IRS1, IRS2, PIK3CD,* |
| KEGG:04910 | **Insulin signaling pathway** | *1,32E-03* | *3,63E-03* | 4,38 | 6 | *PIK3R2, TSC1* |
|  | **Phospholipase D signaling** |  |  |  |  | *EGFR, GAB1, PIK3CD,* |
| KEGG:04072 | **pathway** | *1,96E-03* | *3,80E-03* | 4,05 | 6 | *PIK3R2, PRKCA, TSC1* |
|  | **Choline metabolism in** |  |  |  |  | *EGFR, PIK3CD, PIK3R2,* |
| KEGG:05231 | **cancer** | *1,73E-03* | *3,81E-03* | 5,10 | 5 | *PRKCA, TSC1* |
|  | **AGE-RAGE signaling** |  |  |  |  |  |
|  | **pathway in diabetic** |  |  |  |  | *BCL2, PIK3CD, PIK3R2,* |
| KEGG:04933 | **complications** | *1,89E-03* | *3,91E-03* | 5,00 | 5 | *PRKCA, VCAM1* |
|  |  |  |  |  |  | *EGFR, FOXO3, PIK3CD,* |
| KEGG:05213 | **Endometrial cancer** | *1,70E-03* | *4,01E-03* | 6,90 | 4 | *PIK3R2* |
|  | **Regulation of lipolysis in** |  |  |  |  |  |
| KEGG:04923 | **adipocytes** | *1,59E-03* | *4,05E-03* | 7,02 | 4 | *IRS1, IRS2, PIK3CD, PIK3R2* |
|  |  |  |  |  |  | *BCL2, EGFR, PIK3CD,* |
| KEGG:04066 | **HIF-1 signaling pathway** | *2,76E-03* | *4,80E-03* | 4,59 | 5 | *PIK3R2, PRKCA*  *IRS1, IRS2, PIK3CD, PIK3R2,* |
| KEGG:04931 | **Insulin resistance** | *2,65E-03* | *4,87E-03* | 4,63 | 5 | *RPS6KA6* |

| **GO Biological Process Terms (let-7g-5p)** |  | | |
| --- | --- | --- | --- |
| **Term** | **Overlap** | **P-value Adjusted** | **P-value** |
| regulation of transcription by RNA polymerase II (GO:0006357) | 608/2206 | 1,54E-11 | 4,66E-08 |
| regulation of transcription, DNA-templated (GO:0006355) | 617/2244 | 1,66E-11 | 4,66E-08 |
| positive regulation of transcription, DNA-templated (GO:0045893) | 331/1183 | 2,21E-07 | 4,14E-04 |
| extracellular structure organization (GO:0043062) | 78/216 | 1,16E-06 | 1,54E-03 |
| positive regulation of transcription by RNA polymerase II (GO:0045944) | 258/908 | 1,37E-06 | 1,54E-03 |
| Wnt signaling pathway, calcium modulating pathway (GO:0007223) | 21/36 | 2,29E-06 | 2,15E-03 |
| external encapsulating structure organization (GO:0045229) | 77/217 | 2,91E-06 | 2,33E-03 |
| regulation of intracellular signal transduction (GO:1902531) | 135/437 | 6,15E-06 | 4,27E-03 |
| protein phosphorylation (GO:0006468) | 150/496 | 7,07E-06 | 4,27E-03 |
| regulation of cell migration (GO:0030334) | 127/408 | 7,60E-06 | 4,27E-03 |
| neutral amino acid transport (GO:0015804) | 17/28 | 1,02E-05 | 4,55E-03 |
| cation transport (GO:0006812) | 63/174 | 1,07E-05 | 4,55E-03 |
| cellular protein modification process (GO:0006464) | 281/1025 | 1,07E-05 | 4,55E-03 |
| negative regulation of transcription, DNA-templated (GO:0045892) | 262/948 | 1,14E-05 | 4,55E-03 |
| neuron projection morphogenesis (GO:0048812) | 53/140 | 1,24E-05 | 4,65E-03 |
| negative regulation of cell migration (GO:0030336) | 54/144 | 1,42E-05 | 4,98E-03 |
| amino acid transmembrane transport (GO:0003333) | 23/45 | 1,56E-05 | 5,16E-03 |
| regulation of gene expression (GO:0010468) | 292/1079 | 2,13E-05 | 6,64E-03 |
| collagen fibril organization (GO:0030199) | 37/89 | 2,32E-05 | 6,86E-03 |
| regulation of RNA metabolic process (GO:0051252) | 29/65 | 3,65E-05 | 1,02E-02 |
| negative regulation of cellular macromolecule biosynthetic process (GO:2000113) | 159/547 | 4,07E-05 | 1,09E-02 |
| regulation of anatomical structure morphogenesis (GO:0022603) | 46/123 | 6,40E-05 | 1,63E-02 |
| chromatin remodeling (GO:0006338) | 40/103 | 7,14E-05 | 1,74E-02 |
| regulation of sodium ion transmembrane transporter activity (GO:2000649) | 20/40 | 8,33E-05 | 1,95E-02 |
| regulation of cell-substrate junction assembly (GO:0090109) | 13/21 | 8,76E-05 | 1,97E-02 |
| proline transport (GO:0015824) | 6/6 | 1,10E-04 | 2,37E-02 |
| amino acid transport (GO:0006865) | 23/50 | 1,29E-04 | 2,57E-02 |
| JNK cascade (GO:0007254) | 20/41 | 1,29E-04 | 2,57E-02 |
| peptidyl-serine modification (GO:0018209) | 58/169 | 1,33E-04 | 2,57E-02 |
| extracellular matrix organization (GO:0030198) | 93/300 | 1,39E-04 | 2,61E-02 |
| intracellular protein transport (GO:0006886) | 102/336 | 1,66E-04 | 2,98E-02 |
| carboxylic acid transport (GO:0046942) | 25/57 | 1,72E-04 | 2,98E-02 |
| negative regulation of transcription by RNA polymerase II (GO:0000122) | 189/684 | 1,86E-04 | 2,98E-02 |
| neuron projection development (GO:0031175) | 58/171 | 1,90E-04 | 2,98E-02 |
| sodium ion transport (GO:0006814) | 35/90 | 1,91E-04 | 2,98E-02 |
| negative regulation of cell motility (GO:2000146) | 42/114 | 1,91E-04 | 2,98E-02 |
| ATP-dependent chromatin remodeling (GO:0043044) | 20/42 | 1,96E-04 | 2,98E-02 |
| protein modification by small protein conjugation (GO:0032446) | 120/409 | 2,25E-04 | 3,33E-02 |
| phosphatidylinositol biosynthetic process (GO:0006661) | 45/126 | 2,62E-04 | 3,77E-02 |
| regulation of cell-matrix adhesion (GO:0001952) | 27/65 | 2,88E-04 | 4,04E-02 |
| phosphorylation (GO:0016310) | 117/400 | 3,05E-04 | 4,14E-02 |
| stress-activated MAPK cascade (GO:0051403) | 26/62 | 3,10E-04 | 4,14E-02 |
| negative regulation of nucleic acid-templated transcription (GO:1903507) | 133/464 | 3,20E-04 | 4,18E-02 |
| heart development (GO:0007507) | 55/164 | 3,77E-04 | 4,81E-02 |
| cell morphogenesis involved in neuron differentiation (GO:0048667) | 30/76 | 3,94E-04 | 4,91E-02 |
| gene silencing by miRNA (GO:0035195) | 16/32 | 4,25E-04 | 5,15E-02 |
| negative regulation of Ras protein signal transduction (GO:0046580) | 17/35 | 4,38E-04 | 5,15E-02 |
| negative regulation of translation (GO:0017148) | 34/90 | 4,41E-04 | 5,15E-02 |

**GO Molecular Function Terms (let-7g-5p)**

| **Term** | **Overlap P-value Adjusted P-value** | | |
| --- | --- | --- | --- |
| GTPase activator activity (GO:0005096) | 116/336 | 5,83E-08 | 6,76E-05 |
| RNA polymerase II transcription regulatory region sequence-specific DNA binding (GO:0000977) | 375/1359 | 1,65E-07 | 9,60E-05 |
| RNA polymerase II cis-regulatory region sequence-specific DNA binding (GO:0000978) | 322/1149 | 2,77E-07 | 1,07E-04 |
| cis-regulatory region sequence-specific DNA binding (GO:0000987) | 320/1149 | 5,65E-07 | 1,64E-04 |
| protein serine/threonine kinase activity (GO:0004674) | 109/344 | 1,39E-05 | 2,74E-03 |
| organic anion transmembrane transporter activity (GO:0008514) | 54/144 | 1,42E-05 | 2,74E-03 |
| mRNA 3'-UTR binding (GO:0003730) | 36/85 | 1,83E-05 | 3,03E-03 |
| GTPase regulator activity (GO:0030695) | 78/233 | 2,81E-05 | 4,08E-03 |
| mRNA binding (GO:0003729) | 84/263 | 9,40E-05 | 1,17E-02 |
| neutral amino acid transmembrane transporter activity (GO:0015175) | 17/32 | 1,08E-04 | 1,17E-02 |
| nuclear localization sequence binding (GO:0008139) | 14/24 | 1,16E-04 | 1,17E-02 |
| ankyrin binding (GO:0030506) | 12/19 | 1,26E-04 | 1,17E-02 |
| MAP kinase phosphatase activity (GO:0033549) | 9/12 | 1,31E-04 | 1,17E-02 |
| MAP kinase tyrosine/serine/threonine phosphatase activity (GO:0017017) | 8/10 | 1,53E-04 | 1,19E-02 |
| protein tyrosine/threonine phosphatase activity (GO:0008330) | 8/10 | 1,53E-04 | 1,19E-02 |
| guanyl-nucleotide exchange factor activity (GO:0005085) | 52/149 | 1,79E-04 | 1,26E-02 |
| DNA-binding transcription activator activity, RNA polymerase II-specific (GO:0001228) | 101/333 | 1,84E-04 | 1,26E-02 |
| small GTPase binding (GO:0031267) | 59/175 | 2,02E-04 | 1,30E-02 |
| acylglycerol O-acyltransferase activity (GO:0016411) | 12/20 | 2,51E-04 | 1,54E-02 |
| transcription cis-regulatory region binding (GO:0000976) | 154/549 | 3,41E-04 | 1,98E-02 |
| carboxylic acid transmembrane transporter activity (GO:0046943) | 24/57 | 4,84E-04 | 2,68E-02 |
| transition metal ion binding (GO:0046914) | 127/445 | 5,21E-04 | 2,74E-02 |
| L-proline transmembrane transporter activity (GO:0015193) | 6/7 | 6,24E-04 | 3,15E-02 |
| GTPase binding (GO:0051020) | 64/201 | 6,57E-04 | 3,18E-02 |
| protein tyrosine/serine/threonine phosphatase activity (GO:0008138) | 12/22 | 8,29E-04 | 3,84E-02 |
| PDZ domain binding (GO:0030165) | 25/63 | 1,06E-03 | 4,72E-02 |
| alanine transmembrane transporter activity (GO:0022858) | 8/12 | 1,10E-03 | 4,72E-02 |

**Top 20 KEGG Terms for the intersected targets of let-7g-5p**

**ID**

**Term**

**p-value**

**(Corrected with Benjamini-**

**p-value Hochberg)**

**% of**

**Number**

**Associated of**

**Genes**

**Genes Associated Genes Found**

| KEGG:04740 | **Olfactory transduction** | *8,10E-29* | *2,34E-26* | 4,06 | *ARRB1, CALM1, CAMK2D, GNAL, GRK3, OR13A1, OR13G1, OR14J1, OR4N4,*  18 *OR51E1, OR52A5, OR52H1, OR5B12, OR8J1, PDE1A, PDE2A, PRKACB, SLC8A2* |
| --- | --- | --- | --- | --- | --- |
| KEGG:03010 | **Ribosome** | *7,60E-06* | *1,10E-03* | 8,86 | *MRPL19, MRPL35, MRPS11, RPL13, RPL15, RPL22L1, RPL32, RPL37, RPL37A,*  14 *RPL38, RPL4, RPLP0, RPS15A, UBA52* |

*AKT2, AKT3, ANGPT1, ANGPT4, ARRB1, BDNF, BRAF, CACNA1D, CACNA1I, CACNA2D3, CACNB2, CACNB4, CACNG1, CACNG4, CASP3, CDC25B, CDC42, CHUK, CRK, CRKL, DUSP1, DUSP16, DUSP2, DUSP3, DUSP4, DUSP5, DUSP6, DUSP7, DUSP9, EFNA3, ELK4, EPHA2, ERBB4, FAS, FASLG, FGF5, FGFR1, FGFR2, FGFR3, FGFR4, FLNA, FLNC, FLT1, GNG12, HGF, INSR, KIT, MAP2K4, MAP2K6, MAP2K7, MAP3K1, MAP3K13, MAP3K2, MAP3K3, MAP3K4, MAP3K7, MAP4K2, MAP4K3, MAP4K4, MAPK1, MAPK11, MAPK8, MAPK8IP1, MAPK8IP2, MAPK9, MAPT, MAX, MEF2C, MRAS, NF1, NFATC1, NFATC3, NGF, NLK, NRAS, NTRK2, PAK1, PAK2, PDGFB, PLA2G4E, PPP3CA, PPP3R1, PRKACB, PTPN7, RASGRF1, RASGRF2, RASGRP1, RASGRP2, RPS6KA3, SOS2, STK4, TAB2,*

KEGG:04010 **MAPK signaling pathway** *3,40E-05 1,97E-03* 32,99 97 *TAOK1, TGFBR1, TGFBR2, TP53, TRAF6*

*ABLIM1, ABLIM3, ARHGEF12, BMP7, BMPR2, CAMK2D, CDC42, CFL2, DCC, EFNA3, EFNB3, ENAH, EPHA1, EPHA2, EPHA3, EPHA4, EPHA7, EPHB1, FZD3, LIMK2, LRIG2, LRRC4C, MAPK1, NEO1, NFATC2, NFATC3, NRAS, NTN1, NTN4, PAK1, PAK2, PAK6, PARD6B, PARD6G, PIK3CA, PLXNA1, PLXNA2, PLXNA4, PLXNC1, PPP3CA, PPP3R1, PTCH1, PTK2, RGMA, ROCK1, ROCK2, SEMA3D, SEMA3F, SEMA4C, SEMA4D, SEMA4F, SEMA4G, SEMA5A, SEMA5B, SEMA6A, SRGAP1, SRGAP2, SRGAP3, SSH1, SSH2, SSH3, TRPC5, TRPC6, UNC5A,*

| KEGG:04360 | **Axon guidance**  **Systemic lupus** | *2,19E-05* | *2,11E-03* | 36,26 | 66 *UNC5C, WNT4*  *CD28, CD80, CD86, GRIN2A, GRIN2B, H2AJ, H2AZ2, H2BC18, HLA-DPA1, HLA-* |
| --- | --- | --- | --- | --- | --- |
| KEGG:05322 | **erythematosus** | *2,96E-05* | *2,14E-03* | 8,82 | 12 *DQA1, IL10, RO60* |
|  | **Staphylococcus aureus** |  |  |  |  |
| KEGG:05150 | **infection** | *3,21E-04* | *1,54E-02* | 8,33 | 8 *DEFB1, HLA-DPA1, HLA-DQA1, IL10, ITGB2, MASP1, PTAFR, SELP* |
|  |  |  |  |  | *CDC40, CDC5L, DDX42, FUS, HNRNPA3, HNRNPC, ISY1, LSM8, PRPF38B,* |
| KEGG:03040 | **Spliceosome** | *5,24E-04* | *1,68E-02* | 11,26 | 17 *PRPF40A, SF3A1, SF3A3, SF3B3, SNRNP200, SRSF2, TCERG1, THOC2* |

*ACTB, AKT2, AKT3, ANK1, ANK3, ARHGEF12, BRAF, CAMK2D, CASP3, CBL, CCND1, CDC42, CDKN1A, COL1A1, COL1A2, DROSHA, ERBB4, FAS, FASLG, FGFR1, FLNA, FLNC, FRS2, FZD3, FZD4, FZD5, GPC3, HBEGF, HGF, HOXD10, HPSE, HPSE2, HSPB2, ITGA2, ITGA5, ITGB3, ITPR3, MAPK1, MAPK11, MDM2, MRAS, MSN, NRAS, PAK1, PDCD4, PIK3CA, PPP1R12B, PRKACB, PTCH1, PTK2, RDX, ROCK1, ROCK2, RPS6KB1, RPS6KB2, SDC2, SMAD2, SOS2, THBS1,*

| KEGG:05205 | **Proteoglycans in cancer** | *4,84E-04* | *1,75E-02* | 33,17 | 68 *TIAM1, TLR4, TP53, VAV3, WNT3, WNT4, WNT7A, WNT8B, WNT9A* |
| --- | --- | --- | --- | --- | --- |
|  |  |  |  |  | *AKT2, AKT3, CASP3, CCND1, CDC42, COL1A1, COL1A2, COL3A1, COL4A1,* |

**AGE-RAGE signaling pathway in diabetic**

*COL4A2, COL4A3, COL4A4, COL4A5, COL4A6, CYBB, EDN1, FOXO1, IL6, MAPK1, MAPK11, MAPK8, MAPK9, NFATC1, NOX4, NRAS, PIK3CA, PLCB1, PLCB2, PLCB3, PLCB4, PLCD4, PRKCE, SMAD2, SMAD3, SMAD4, TGFBR1,*

KEGG:04933 **complications** *4,37E-04 1,80E-02* 38,00 38 *TGFBR2, VCAM1*

|  | | | | | *ACVR1B, ACVR1C, ACVR2A, ACVR2B, BMP2, BMP5, BMP7, BMPR1A, BMPR2, CDKN2B, CHRD, E2F5, FBN1, FMOD, GDF6, GREM1, LEFTY1, MAPK1, NEO1,* |
| --- | --- | --- | --- | --- | --- |
|  | **TGF-beta signaling** |  |  |  | *PPP2R1B, RGMA, ROCK1, RPS6KB1, RPS6KB2, SKP1, SMAD2, SMAD3, SMAD4,* |
| KEGG:04350 | **pathway** | *6,81E-04* | *1,97E-02* | 38,30 | 36 *SMAD5, SMAD7, SP1, TGFBR1, TGFBR2, THBS1, THSD4, ZFYVE16* |
|  |  |  |  |  | *ACVR1B, ACVR1C, ACVR2A, ACVR2B, AKT2, AKT3, APC, APC2, BMPR1A,* |
|  |  |  |  |  | *BMPR2, DUSP9, DVL3, FGFR1, FGFR2, FGFR3, FGFR4, FZD3, FZD4, FZD5,* |

**Signaling pathways regulating pluripotency of**

*HAND1, HOXB1, HOXD1, IL6ST, ISL1, JARID2, KAT6A, LEFTY1, LIF, LIFR, MAPK1, MAPK11, MEIS1, NRAS, PAX6, PCGF3, PIK3CA, RIF1, SETDB1, SKIL, SMAD2, SMAD3, SMAD4, SMAD5, SMARCAD1, WNT3, WNT4, WNT7A, WNT8B,*

KEGG:04550 **stem cells** *7,84E-04 2,06E-02* 34,97 50 *WNT9A, ZFHX3*

|  | | | | | *ABI2, ACTB, APC, APC2, ARHGAP35, ARHGEF12, ARHGEF6, ARPC5, BRAF, CDC42, CFL2, CHRM3, CHRM5, CRK, CRKL, DIAPH2, DIAPH3, DOCK1, ENAH, FGF5, FGFR1, FGFR2, FGFR3, FGFR4, GNG12, IQGAP2, IQGAP3, ITGA1, ITGA10, ITGA11, ITGA2, ITGA5, ITGA7, ITGA8, ITGB2, ITGB3, ITGB6, ITGB7, KNG1, LIMK2, MAPK1, MRAS, MSN, MYH10, MYLK, MYLK3, MYLK4, NCKAP1L,* |
| --- | --- | --- | --- | --- | --- |
|  | **Regulation of actin** |  |  |  | *NRAS, PAK1, PAK2, PAK6, PDGFB, PIK3CA, PIP4K2C, PPP1R12B, PTK2, RDX,* |
| KEGG:04810 | **cytoskeleton** | *1,75E-03* | *4,22E-02* | 31,65 | 69 *ROCK1, ROCK2, SOS2, SPATA13, SSH1, SSH2, SSH3, TIAM1, VAV3, VCL, WASL* |
|  | **Neutrophil extracellular** |  |  |  | *ACTB, AKT2, AKT3, CLCN5, CYBB, H2AJ, H2AZ2, H2BC18, HDAC11, HDAC2, HDAC9, ITGB2, ITGB3, MAP3K7, MAPK1, MAPK11, PADI4, PIK3CA, PLCB1,* |
| KEGG:04613 | **trap formation** | *2,09E-03* | *4,31E-02* | 13,68 | 26 *PLCB2, PLCB3, PLCB4, SELP, SLC25A4, SYK, TLR4* |
|  |  |  |  |  | *ADCY1, ADCY9, ADRB1, ADRB2, ADRB3, AKT2, AKT3, ATF6B, ATP2A2, ATP2A3,* |
|  |  |  |  |  | *ATP2B1, ATP2B2, ATP2B3, ATP2B4, CACNA1D, CALM1, CREB3L2, CREB3L4,* |
|  |  |  |  |  | *CREB5, GNAQ, GTF2I, GTF2IRD1, GUCY1A1, GUCY1A2, INSR, IRS1, IRS2,* |
|  |  |  |  |  | *ITPR3, KCNMA1, KCNMB4, KNG1, MAPK1, MEF2C, MEF2D, MYLK, MYLK3,* |
|  | **cGMP-PKG signaling** |  |  |  | *MYLK4, NFATC1, NFATC2, NFATC3, PDE2A, PDE5A, PIK3R5, PLCB1, PLCB2, PLCB3, PLCB4, PPP3CA, PPP3R1, PRKCE, ROCK1, ROCK2, SLC25A4, SLC8A2,* |
| KEGG:04022 | **pathway** | *1,94E-03* | *4,32E-02* | 32,93 | 55 *TRPC6* |

*ACTB, AMOT, APC, APC2, BMP2, BMP5, BMP7, BMPR1A, BMPR2, BTRC, CCND1, CCND2, CDH1, CRB1, CRB2, CSNK1E, DLG2, DVL3, FZD3, FZD4, FZD5, GDF6, GLI2, ITGB2, LIMD1, LLGL2, MOB1A, NKD1, PARD6B, PARD6G, PATJ, PPP2R1B, PPP2R2A, SCRIB, SMAD2, SMAD3, SMAD4, SMAD7, TCF7L1, TEAD1, TEAD3, TGFBR1, TGFBR2, WNT3, WNT4, WNT7A, WNT8B, WNT9A, WWTR1,*

KEGG:04390 **Hippo signaling pathway** *3,77E-03 4,74E-02* 32,48 51 *YAP1, YWHAZ*

*AKT2, AKT3, APAF1, BAK1, BCL2L1, CASP3, CCND1, CCNE2, CDK6, CDKN1A, CDKN2B, CHUK, COL4A1, COL4A2, COL4A3, COL4A4, COL4A5, COL4A6, E2F2, ITGA2, LAMA1, LAMC1, LAMC2, MAX, PIK3CA, PTGS2, PTK2, RB1, SKP2, TP53,*

| KEGG:05222 | **Small cell lung cancer** | *3,66E-03* | *4,80E-02* | 35,87 | 33 *TRAF3, TRAF5, TRAF6* |
| --- | --- | --- | --- | --- | --- |
| KEGG:02010 | **ABC transporters** | *3,50E-03* | *4,82E-02* | 42,22 | *ABCA12, ABCA13, ABCA2, ABCA5, ABCB10, ABCB5, ABCB9, ABCC1, ABCC10,*  19 *ABCC5, ABCC6, ABCC8, ABCC9, ABCD2, ABCD4, ABCG4, ABCG8, CFTR, DEFB1* |
|  | **Metabolism of xenobiotics** |  |  |  |  |
| KEGG:00980 | **by cytochrome P450** | *2,51E-03* | *4,83E-02* | 8,97 | 7 *AKR1C1, CYP1B1, CYP2F1, GSTO2, MGST2, UGT1A1, UGT2A1* |
